# Water‐Enhanced Multicolor Electrochromism in Nickel‐Catecholate MOFs

**DOI:** 10.1002/advs.202500678

**Published:** 2025-03-27

**Authors:** Qi Zhao, Jing Yang, Xingyang Wang, Wanwan Wang, Yulin Gao, Xue Chen, Jianguo Sun, Hao Yuan, Yu Liu, Jinwoo Park, Lewis Kien Juen Ting, Qing Wang, Pooi See Lee, Yanfeng Gao, Yong‐Wei Zhang, John Wang

**Affiliations:** ^1^ Department of Materials Science and Engineering National University of Singapore Singapore 117575 Republic of Singapore; ^2^ Institute of High Performance Computing (IHPC) Agency for Science Technology and Research (A*STAR) 1 Fusionopolis Way, #16‐16 Connexis Singapore 138632 Republic of Singapore; ^3^ Institute of Materials Research and Engineering (IMRE) Agency for Science Technology and Research (A*STAR) 2 Fusionopolis Way, Innovis #08‐03 Singapore 138634 Republic of Singapore; ^4^ School of Materials Science and Engineering Shanghai University Shanghai 200444 P. R. China; ^5^ School of Materials Science and Engineering Nanyang Technological University 50 Nanyang Avenue Singapore 639798 Republic of Singapore; ^6^ School of Chemical and Environmental Engineering Anhui Polytechnic University Wuhu 241000 P. R. China; ^7^ National University of Singapore (Chongqing) Research Institute Chongqing Liang Jiang New Area Chongqing 401120 P. R. China

**Keywords:** coloring mechanism, conducting metal–organic frameworks, multicolor electrochromic behavior, water molecules

## Abstract

Metal–organic frameworks (MOFs) represent a novel electrochromic material system but are limited in tunable color versatility, rapid switching speed, and long‐term cycling stability. A new multicolor electrochromic behavior is reported, with transitions from green to blue to purple, in conducting nickel‐catecholate (Ni‐CAT‐1) MOFs. The system significantly improves cycling stability to 2000 cycles and reduces switching time to 3.6 s, both of which outperform most state‐of‐the‐art MOF systems. An in‐depth understanding of the coloring mechanism, particularly the interconversion among C─O, C─O^•^, and C = O groups is revealed. Water molecules play diverse roles in this redox process. Specifically, water molecules induce distortions in Ni─O bonds, facilitating C = O bond formation and expediting the oxidized coloring process, while also aiding the dissociation of ions from solvation complexes to enhance the reduction process. The practical applications of these findings are demonstrated by designing flexible multicolor electrochromic devices (FMEDs) for use in camouflage, flexible displays, and augmented reality (AR).

## Graphical abstract

This work reports a new multicolor electrochromic behavior in conducting nickel‐catecholate (Ni‐CAT‐1) MOFs, exhibiting transitions from green to blue and then to purple. The coloring mechanism involves the interconversion of C─O, C─O^•^, and C = O bonds. Additionally, water molecules induce distortions between Ni─O, promoting C = O bond formation while assisting in ion desolvation and diffusion.

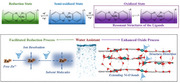

## 1 Introduction

Color‐functional technologies have wide‐ranging applications in optical technologies, sensing and monitoring, and aesthetic design.^[^ ^1^ ^]^ Among them, multicolor electrochromic technology has been extensively incorporated into the field of flexible electronics, distinguished by its unique tunable optical properties, low threshold voltage, and minimal power consumption.^[^ ^2^ ^]^ These attributes are particularly advantageous for applications such as flexible displays, wearable and portable electronics, adaptive camouflage, and intelligent optics.^[^ ^3^ ^]^ Achieving multicolor properties typically involves the use of conducting polymers, such as polyaniline and viologen, or constructing Fabry‐Pérot structures with inorganic materials.^[^ ^4^ ^]^ Compared to these conventional material systems, metal‐organic frameworks (MOFs) provide more accessible active sites, more tunable porous structures, and much higher surface areas (up to 10000 m^2^ g^−1^). Theoretically, these advantages are favorable for color switching.^[^ ^5^ ^]^ Particularly, since the pioneering work on MOF‐74 reported by the Dincă group, HKUST‐1, NBU‐3, and their derivatives have been demonstrated as electrochromic materials.^[^ ^6^ ^]^ However, most of these MOFs display only two colors. Taking naphthalene diimide (NDI) linkers as a typical example, the presence of only two redox states, [NDI]^•‐^ and [NDI],^2^ ^−^ limits their multicolor characteristics.^[^ ^7^ ^]^ Moreover, common issues such as slow switching speed (>10 s) and unsatisfactory cycling performance (<100 times) remain unresolved.^[^ ^6^, ^8^ ^]^

Recently, we have observed that the conductive MOFs (c‐MOFs), as a subset, offer advantages that surpass those of traditional MOFs and hold promising great potential to address the aforementioned issues.^[^ ^9^ ^]^ The conjugated ligands in c‐MOFs typically serve as redox‐active ligands capable of reversible interconversions between multitype redox states, establishing a robust basis for newly discovered multicolor transitions.^[^ ^10^ ^]^ For example, 2,3,6,7,10,11‐hexahydroxytriphenylene (H_12_C_18_O_6_, HHTP) can transit between the catecholate, semiquinonate, and quinone forms.^[^ ^11^ ^]^ Moreover, the extended π–π and π‐d orbital overlaps lead to increased conductivity, ranging from 10^−4^ to 10^−1^ S cm^−2^,^[^ ^5^, ^10^, ^12^ ^]^ further facilitating electron transport and stabilizing charged/discharged states during electrochemical reactions.^[^ ^13^ ^]^ Despite significant advances in the use of c‐MOFs for supercapacitors,^[^ ^14^ ^]^ batteries,^[^ ^5b^ ^]^ and catalysis,^[^ ^15^ ^]^ research into their electrochromic behavior remains rather limited. One underlying reason is the ambiguous effects of the constituted chemical and ligand groups within the internal structure and the unknown coloring mechanism. This prompts inquiries into the structural configuration of ligands during different stages of the redox process and their consequent impact on the dynamic characteristics of metal‐oxygen bonds, as well as the factors influencing coloration.

Herein, we demonstrate for the first time a three‐color switching in Ni‐catecholates (Ni‐CAT‐1) c‐MOFs, transitioning from green to blue and then to purple. The new coloring mechanism is based on the interconversion of C─O, C─O, and C = O bonds, accompanied by changes in the coordination environment of the central Ni node. Furthermore, by combining experimental and computational studies, we gain new insights into the interactions between the central metal ions and organic ligands in the presence of water molecules, extending beyond their commonly recognized role in maintaining structural stability through hydrogen bonding.^[^ ^11^, ^16^, ^17^ ^]^ During the oxidation process, water molecules extend Ni‐O bond lengths and induce distortions, further promoting the formation of C = O bonds. Conversely, during the reduction process, water molecules assist in the dissociation of ions from the solvation complex structure, facilitating ion diffusion. With the help of water, the switching speed is significantly enhanced (coloration/bleaching time: 3.6/4.2 vs 4.6/6.6 s) and the cycling stability is greatly improved (2000 vs 20 cycles) compared to dehydrated samples. As a proof‐of‐concept demonstration, flexible multicolor electrochromic devices (FMEDs) assembled with Ni‐CAT‐1 are proposed for various applications, including flexible displays, wearable electronics, and camouflage devices.

## 2 Result and Discussion

### 2.1 Phase Characterization and Morphology

**Figure 1:**
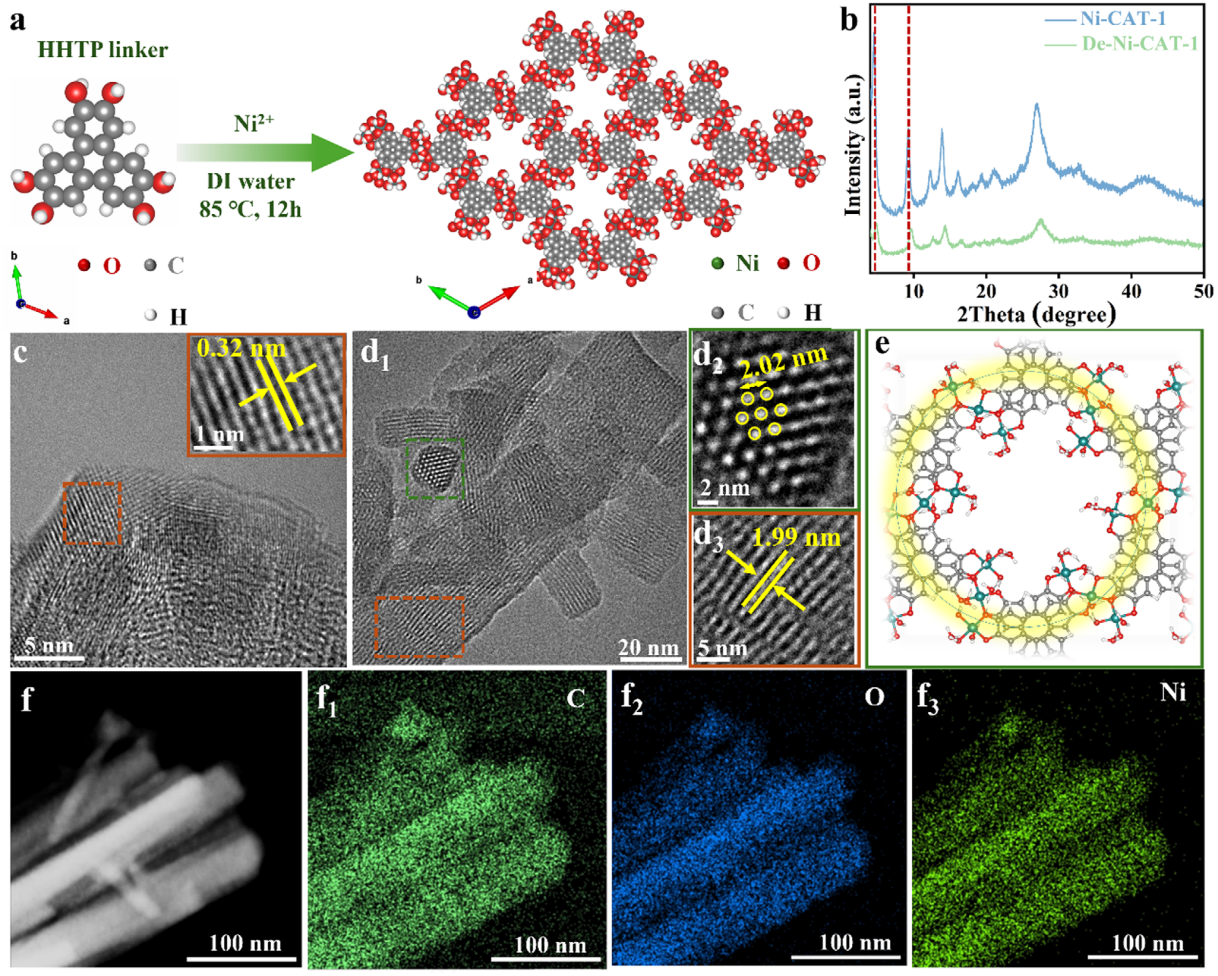
Phase characterization and morphology of Ni‐CAT‐1. a) Synthetic diagram and molecular packing of Ni‐CAT‐1. b) XRD patterns of Ni‐CAT‐1 and De‐Ni‐CAT‐1. c,d) cryo‐TEM images of Ni‐CAT‐1 conducted at −165 °C. (d_1_‐d_3_) Inverse FFT images of (d_1_), with honeycomb structure highlighted in green (d_2_) and lattice stripes in orange (d_3_). e) Corresponding schematic structure of (d_2_). f) EDS elemental mapping results of Ni‐CAT‐1.

The Ni‐CAT‐1 was synthesized via a typical hydrothermal process, in which the organic ligand HHTP reacted with Ni^2+^ ions in deionized water at 85 °C (**Figure** **1**a). Typically, the structure of Ni‐CAT‐1 features two distinct types of alternatively stacked layers (Figure 1a; Figure S1, Supporting Information): one layer consists of extended honeycomb‐like structures (Figure S2, Supporting Information), while the other comprises discrete units (Figure S3, Supporting Information).^[^ ^11^ ^]^ Within these layers, each metal atom is coordinated to two or four water molecules, respectively, thus contributing to the structural stability through hydrogen bonding.^[^ ^11^ ^]^ Achieving a perfect match between the coordination numbers of water molecules and central metal ions presents challenges.^[^ ^10b^ ^]^ Redundant water molecules can be absorbed within the layers due to the solvent impurities and environmental humidity. Considering this, a comparison sample of dehydrated Ni‐CAT‐1 (De‐Ni‐CAT‐1) treated with chloroform was prepared in order to fully understand the effect of water molecules. Simultaneous differential scanning calorimetry (DSC) and thermogravimetric analysis (TGA) reveal a large endotherm associated with dehydration in both samples but with the chloroform‐treated sample exhibiting a substantially smaller peak height (Figure S4, Supporting Information). Mass loss based on the dehydration endotherm onset and offset temperatures shows the water content decreases from 26% to 22% after treatment, corresponding to a reduction in the number of water molecules coordinated to each nickel atom from 4.3 to 3.4. This indicates the removal of a significant amount of adsorbed water following chloroform treatment. Furthermore, Powder X‐ray diffraction patterns (PXRD) analysis reveals that Ni‐CAT‐1 and De‐Ni‐CAT‐1 have almost identical diffraction patterns (Figure 1b), featuring typical planes of are (100), (200), (210), and (001).^[^ ^15a^ ^]^ However, the peaks of De‐Ni‐CAT‐1 shift to larger theta degrees, indicating a decrease in the interlayer spacing of NiCAT‐1 due to the removal of absorbed water from the interlayer. Further compositional details of Raman spectroscopy and Fourier transform infrared (FTIR) spectra are provided in Figure S5 and Note S1 (Supporting Information). To prevent the structural damage of Ni‐CAT‐1 from the high‐energy beam, cryo‐transmission electron microscopy (TEM) was conducted at −165 °C (Figure S6, Supporting Information). The images show a nanorod‐like morphology with average lengths ranging from 200 to 1000 nm and average diameters ranging from 20 to 100 nm (Figures S7 and S8, Supporting Information). Typically, the lattice spacing of 0.32 nm is attributed to the interplanar distance of (001) plane, indicating π‐π stacking (Figure 1c). Another typical crystal plane (2θ = 4.6 °) is (100), corresponding to the lattice spacing of 1.99 nm (Figure 1d_3_; Figure S8, Supporting Information). Ordered and spacious channels/pores (≈2.02 nm) in a honeycomb arrangement, viewed along the [001] direction, facilitate the ion diffusion process (Figure 1d,e). Corresponding EDS results are used to verify the C, O, and Ni elements in the Ni‐CAT‐1 (Figure 1f). On the other hand, De‐Ni‐CAT‐1 shows very similar morphology and lattice details, as seen in Figures S9–S12 and Note S2 (Supporting Information). Such subtle morphological differences arise from hydrogen bonding interactions between water molecules and the MOF framework. Additionally, water complexation with central metal ions alters their coordination number and geometric structure. Notably, conductivity measurements on a compressed pellet were conducted using a four‐point probe measured at room temperature (Note S3, Supporting Information). Electrical conductivities of the Ni‐CAT‐1 and De‐Ni‐CAT‐1 are measured to be 3.75 × 10^−4^ and 2.88 × 10^−4 ^S cm^−1^, respectively, which match well with the previously reported values.^[^ ^5b^ ^]^ Such good electrical conductivity can enhance the charge transfer. These results not only confirm the successful synthesis of Ni‐CAT‐1 but also indicate that the dehydration process does not significantly alter the phase structure of Ni‐CAT‐1; rather, it solely removes absorbed water within the MOF structure.

### 2.2 Electrochromic and Electrochemical Performance

**Figure 2:**
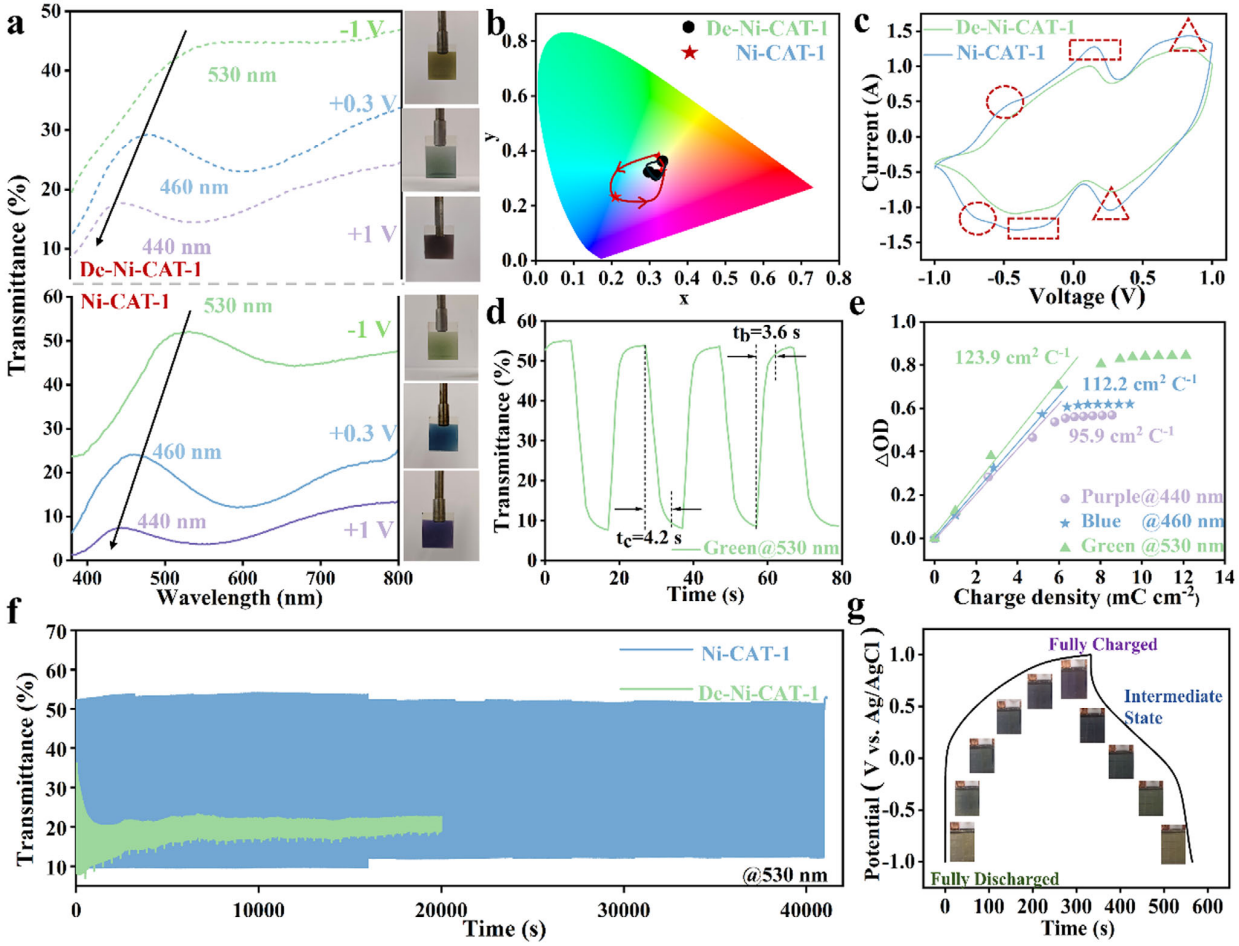
Multicolor and electrochemical properties. a) In situ transmittance spectra (800–380 nm) of Ni‐CAT‐1 and De‐Ni‐CAT‐1 at ‐1 V, +0.3 V, and +1 V, along with corresponding digital photos of multicolor transitions. b) Color coordinates of Ni‐CAT‐1 and De‐Ni‐CAT‐1 at −1, +0.3, and +1 V mapped in CIE 1931 chromaticity diagram. c) Cyclic voltammetry curves at 10 mV s^−1^. d) Switching speeds of Ni‐CAT‐1 at 530 nm. e) Coloration efficiency of Ni‐CAT‐1 at different wavelengths. f) Cycling performance of Ni‐CAT‐1 and De‐Ni‐CAT‐1 with an interval time of 20 s. g) Galvanostatic charge/discharge curves of Ni‐CAT‐1 at a current density of 0.3 mA cm^−2,^ with inserts showing the corresponding color evolution.

To further investigate the effect of water on electrochemical behavior, we conducted a series of electrochromic and electrochemical experiments. Ni‐CAT‐1 and De‐Ni‐CAT‐1 electrodes were fabricated via a spray‐coating method (see Section S1.3, Supporting Information), followed by examination using a three‐electrode system in a 0.2 m Zn(ClO_4_)_2_/propylene carbonate electrolyte. A standard Ag/AgCl electrode served as the reference electrode, while a platinum plate was utilized as the counter electrode. Film thickness plays a crucial role in electrochromic performance, including switching speed, optical modulation, cycling stability, and coloration efficiency. Thinner films have fewer active materials, resulting in lower optical modulation but faster switching speed due to shorter ion diffusion pathways. In contrast, thicker films, particularly those prepared by spraying, exhibit higher surface roughness and uneven electric field distribution, potentially compromising cycling stability. To isolate the effect of water molecules, we maintain consistent film thickness between the two samples (Figure S13, Supporting Information). As a result, *in‐situ* optical transmittance spectra of the Ni‐CAT‐1 electrode demonstrate a blue shift from 530 to 460 nm to 440 nm at potentials of −1 V, +0.3 V, and +1 V (vs Ag/AgCl), resulting in transitions from green to blue and then to purple, respectively (**Figure** **2**a; Video S1, Supporting Information). The corresponding direct optical bandgaps (*E_g_*) for different potential ranges are determined by fitting the Tauc plots calculated from the absorbance spectra, as shown in Figure S14 and Note S4 (Supporting Information). The *E_g_* values are 2.40, 2.68, and 2.55 eV at potentials of −1 V, +0.3 and +1 V (vs Ag/AgCl), respectively, aligning with color‐switching phenomena. Although the De‐Ni‐CAT‐1 exhibits a similar shifting trend, the color distinction is less pronounced, as evidenced by the inset digital photos. Especially, The *E_g_* values in blue and purple states are close, at 2.76 and 2.73 eV, respectively (Figure S15, Supporting Information). CIE color coordinates provide more evidence, suggesting that the color span range in Ni‐CAT‐1 is larger than that in De‐Ni‐CAT‐1, suggesting better color modulation (Figure 2b). Furthermore, cyclic voltammetry (CV) of Ni‐CAT‐1 exhibits three pairs of redox peaks at −0.52 V/−0.73 V, 0.15 V/−0.40 V, and 0.83 V/0.27 V, respectively, which align with the absorption shift in the optical spectrum (Figure 2c). For comparison, the enclosed area within the CV of Ni‐CAT‐1 is larger than that of De‐CAT‐1, indicating the involvement of more ions/electrons upon applied sweeping voltages.^[^ ^18^ ^]^

In addition to their multicolor properties, our Ni‐CAT‐1 electrodes also demonstrate fast electrochemical kinetics. Quantitatively, the optical contrasts (ΔT) of Ni‐CAT‐1 at 530, 460, and 440 nm are 48.1%, 32.4%, and 26.2%, respectively (Figure 2a), nearly twice those of De‐Ni‐CAT‐1 (24.4%, 13.0% and 12.5%). Coloration and bleaching speeds, defined as the time for a 90% change of the whole ΔT, are 4.2/3.6 s, 6.0/3.0 s and 5.0/4.0 s at 530, 460, and 440 nm, respectively, which are faster than those of De‐Ni‐CAT‐1 (6.6/ 4.6 s, 6.0/4.0 s, and 8.2/ 6.0 s) and previously reported MOF electrodes (>10 s) (Figure 2d; Figures S16 and S17, Supporting Information).^[^ ^8^, ^19^ ^]^

Moreover, coloration efficiency (CE) is defined as the change in optical density per unit of inserted charge.^[^ ^20^ ^]^ Essentially, a higher CE suggests greater optical modulation achieved with less charge consumption.^[^ ^21^ ^]^ As indicated by Figure 2e and Figure S18 (Supporting Information), our CE values are nearly three times higher compared to those of dehydrated samples (123.9 vs 50.0 cm^2^C^−1^ @ 530 nm, 112.2 vs 35.4 cm^2^C^−1^ @ 460 nm, and 95.9 vs 37.3 cm^2^C^−1^ @ 440 nm).

To examine the cycling stability of Ni‐CAT‐1, *in‐situ* changes in transmittance at 530 nm within the potential window of −1 to +1 V were measured (Figure 2f). Ni‐CAT‐1 exhibits a long cycling life, retaining 97.1% of its original optical modulation after 40 000 s (≈2000 cycles), significantly surpassing existing electrochromic MOF electrodes (ranging from 6 to 900 cycles in **Table** **1**). In contrast, De‐Ni‐CAT‐1 maintains only 75% after 400 s (≈20 cycles) and 15.5% after 20 000 s. It should be noted that higher potentials can intensify the effects of uneven electric fields across the electrode, leading to accelerated electrode degradation and structural instability. Interestingly, we performed an extreme experiment by testing Ni‐CAT‐1 in an aqueous electrolyte (Figure S19, Supporting Information). While the switching time was remarkably reduced to 0.9 s, the cycling stability drastically declined to fewer than two cycles. This prompts us to consider the dual roles of water molecules.

**Table 1:** Summary of state‐of‐the‐art electrochromic MOF electrodes and other representative electrochromic materials.

Materials	Applied voltage [V]	Optical modulation [%]	Switching speed [s]	Coloration efficiency [cm^2 ^C^−1^]	Cycling times	Capacity	Color transition
MOF‐74^[^ ^6a^ ^]^	−0.5/−2	/	7/23	100	10	/	Transparent to dark
HKUST‐1^[^ ^6b^ ^]^	−0.4/+0.8	64 @460 nm	5/6	/	100	/	Light blue to bright blue
HKUST‐1^[^ ^19a^ ^]^	−1.5/+0.2	24.7 @500 nm	23/31.9	80.6	900	/	Pale blue to dark blue
ZnMOF‐74^[^ ^6b^ ^]^	−1/+2	31 @600 nm	10/7	/	50	/	Brown to yellow
NiMOF‐74^[^ ^19b^ ^]^	−0.8/+1.2	44.4 @550 nm	24.5/23.5	/	650	1438 µF cm^−2^ @ 50 mV s^−1^	light yellow to reddish brown
ZnTCA^[^ ^27^ ^]^	+0.1/+1.8	57 @702 nm	4.5/6.2	/	150	/	Pale yellow to blue
NBU‐3^[^ ^6c^ ^]^	−2/+2	/	/	/	6	/	Transparent to yellow
Zr‐NDI^[^ ^8a^ ^]^	−2.5/0	29 @470 nm	7.7/43	51.38	<10	/	Light yellow to dark brown
Ni_3_(HITP)_2_^[^ ^28^ ^]^	−1.5/+0.2	15.7 @780 nm	4.3/5.4	100	100	/	Yellow to blue
Zn_2_(PDICl_4_)_2_^[^ ^8b^ ^]^	−0.8/+0.5	/	14	104.8	100	/	Orange to blue
Zn(NDI‐X)^[^ ^8c^ ^]^	−1.75/0	/	/	60‐100	25	/	Green to orange to purple
NU‐901^[^ ^29^ ^]^	0/+1.6	62 @587 nm	5/12	204	20	/	Yellow to blue
[V2O2+ξ(OH)3‐ξ]^[^ ^30^ ^]^	−1.5/+0.5/+2	50 @640 nm	7/14.5	96.6	600	610 Fg^−1^ @ 1 A g^−1^	Red to green to blue
NiO^[^ ^31^ ^]^	−0.5/+0.8	32.2 @550 nm	4.5/5	88.1	10 000	/	Brown dark to transparent
WO_3_^[^ ^21^ ^]^	−1/+1	81.7 @630	6/22	57.7	100	/	Blue to transparent
Ni‐CAT‐1 (This work)	−1/+0.3/+1	48.1 @530 nm	4.2/3.6	123.9	2000	160.9 mAh m^−2^ @ 0.3 mA cm^−2^	Green to blue to purple

Additionally, a unique advantage not attainable by conventional batteries/supercapacitors is real‐time energy monitoring through visual color conversion. As shown in Figure 2g, from purple (+1 V) to green (−1 V), the Ni‐CAT‐1 demonstrates a high areal capacity of 160.9 mAh m^−2^ at a current density of 0.3 mA cm^−2^, which is also higher than those of other known electrochromic energy storage devices, such as TiO_2_ (127.8 mAh m^−2^ at 0.06 mA cm^−2^),^[^ ^22^ ^]^ PB (77.1 mAh m^−2^ at 0.02 mA cm^−2^),^[^ ^23^ ^]^ Nb _18_W_16_O_93_ (106.7 mAh m^−2^ at 0.25 mA cm^−2^),^[^ ^24^ ^]^ WO_3_ (101.1 mAh m^−2^ at 0.25 mA cm^−2^,^[^ ^25^ ^]^126.3 mAh m^−2^ at 1 mA cm^−2^).^[^ ^26^ ^]^ Likewise, transitions from green (−1 V) to blue (+0.3 V) and from blue (+0.3 V) to purple (+1 V) yield high areal capacities of 40.8 and 85.9 mAh m^−2^ at current densities of 0.3 mA cm^−2^, suggesting the potential of visually discernible color changes as energy storage level indicators (Figures S20 and S21, Supporting Information). Specifically, green signifies a fully discharged state, blue indicates an intermediate state of charge, and purple denotes a fully charged state. Although De‐Ni‐CAT‐1 also exhibits energy‐monitoring functionality, their areal capacities are consistently lower than those of Ni‐CAT‐1, with values of 99.5, 51.2, 37.2, 34.5, and 9 mAh m^−2^ at current densities of 0.3, 0.5, 0.8, 1 and 2 mA cm^−2^, respectively (Figures S22 and S23 and Note S5, Supporting Information).

In brief, Table 1 presents key electrochromic and electrochemical indicators, providing a clear overview of the state‐of‐the‐art electrochromic MOF electrodes and other representative electrode materials. Our novel Ni‐CAT‐1 electrode offers comprehensive performance, including multicolor transition, fast switching speed, high stability, and large areal capacity, demonstrating its promise as a candidate for FMEDs.

### 2.3 Diverse Roles of Water Molecules

**Figure 3:**
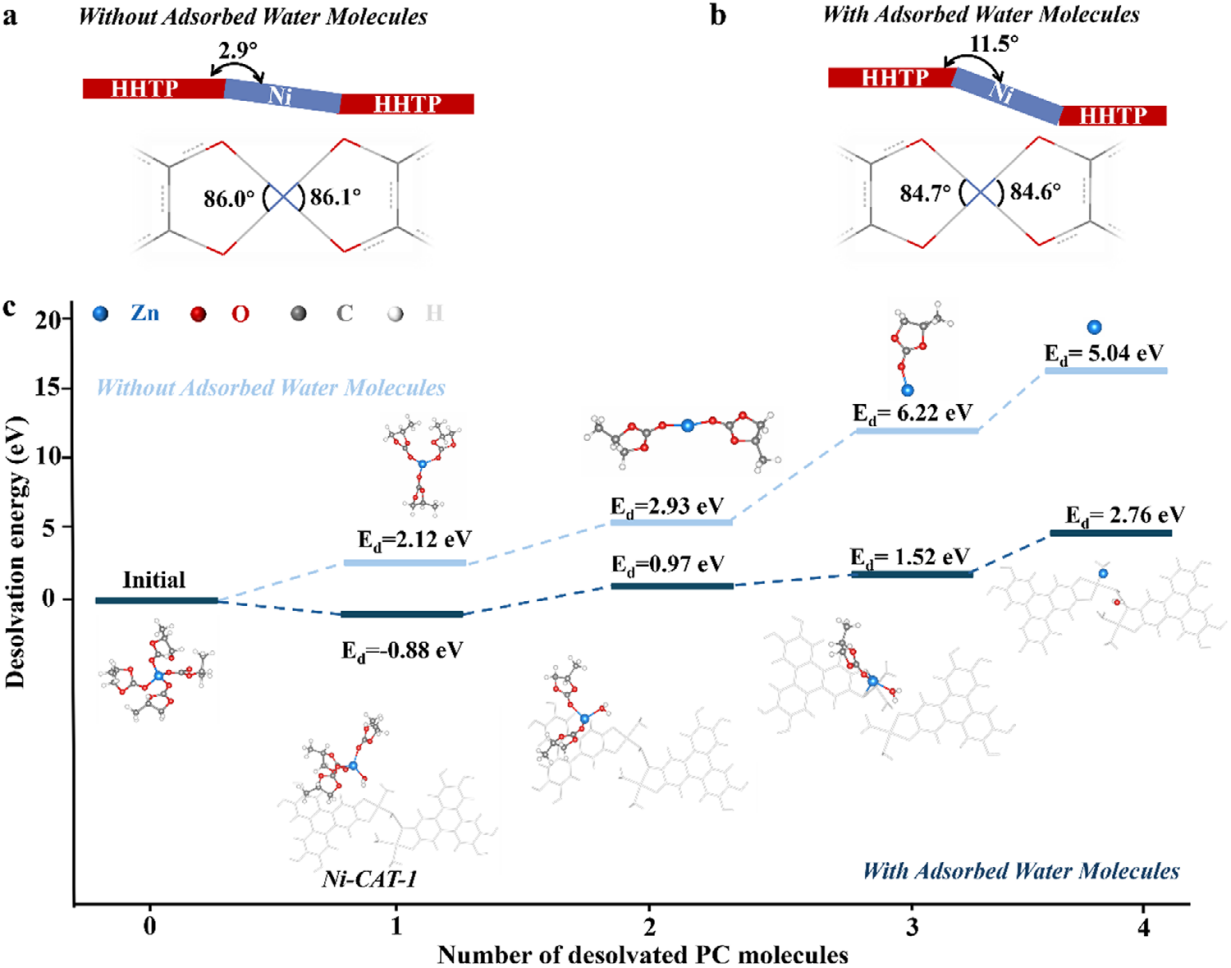
Theoretical understanding of the diverse roles of adsorbed H_2_O. Schematic diagram of the structure of Ni‐MOF without a) and with b) adsorbed water in the unit cell. c) Stepwise desolvation energies of Zn‐PC with (bottom) and without (top) water molecules. Insets: molecular geometries corresponding to the desolvated of Zn(PC)_x_^2+^. The desolvation energy (in eV) for each step is provided accordingly. Blue, red, grey, and white spheres represent Zn, O, C, and H atoms, respectively.

To clearly elucidate the origin of the significant improvement between samples with and without adsorbed water, we provide in‐depth insights into the diverse roles that water molecules play. DFT calculations were performed to investigate the dynamic behaviors of the Ni‐O bonds associated with the structural distortions. The optimized structures are depicted in **Figure** **3**a. Upon increasing the water concentration (Figure 3b), notable distortions are evident, particularly within the region highlighted by the purple box. Quantitative analysis of these distortions, including bond lengths and dihedral angles between the HHTP units is summarized in Table S1 (Supporting Information). With higher water concentrations, there is an elongation of Ni─O bonds (from 185–189 pm to 189–191 pm) and an increase in dihedral angles (from 2.9° to 11.5°). Such distortion makes the conversion of Ni─O ionic bonds to Ni─O coordinate bonds easier, potentially expediting the oxidized coloring process. This process will be further discussed in the following “Coloring Mechanism” section.

In addition, DFT calculations were further conducted to validate the beneficial effects of water molecules on ion‐diffusion kinetics. Specifically, ab initio molecular dynamics calculations suggest four oxygen atoms were involved in the first coordination layer of Zn^2^⁺ (Figure S24, Supporting Information), indicating the PC coordinated Zn^2+^ in the form of Zn(PC)_4_^2+^. Similar findings were also reported for the coordination of PC with Li⁺.^[^ ^32^ ^]^ The desolvation energies of Zn(PC)_4_^2+^ with and without the assistance of H₂O are summarized in Figure 3c and Table S2 (Supporting Information), and the detailed desolvation mechanism of Zn(PC)_4_^2+^ is detailed in the computational section. As shown in Figure 3c and H₂O significantly facilitates the desolvation from Zn(PC)_4_^2+^ to Zn^2+^ by effectively lowering the desolvation energy. The initial step involves substituting one PC molecule. In the absence of H₂O, the reaction energy required to remove the first PC was found to be 2.12 eV. However, in the presence of H_2_O, the H₂O molecule replaces one PC in Zn(PC)_4_^2+^, resulting in Zn(PC)_3_(H_2_O)^2+^. The entire process is exothermic with an energy release of 0.88 eV. The introduction of H₂O into the Zn‐PC cluster weakens the interaction between Zn^2^⁺ and the PC molecules, thereby reducing the reaction energies required to remove the second, third, and fourth PC molecules. As summarized in Table S2 (Supporting Information), the energies required to remove the subsequent PC molecules in the absence of H_2_O are 2.93, 6.22, and 5.04 eV, respectively. However, in the presence of H_2_O, these energies are reduced to 0.97, 1.52, and 2.76 eV. Overall, H_2_O effectively facilitates the desolvation from Zn(PC)_4_^2+^ to Zn^2+^, thereby assisting in free zinc ion diffusion.

### 2.4 Coloring Mechanism

Although it is commonly accepted that the redox process involves the interconversion of multiple redox states between catecholate, semiquinonate, and quinone, the precise chemical states of these formations remain ambiguous.^[^ ^33^ ^]^ Furthermore, it is unclear whether the valence of the central metal ion changes during the redox reaction.^[^ ^5^, ^28^ ^]^ Based on our investigation into the significant effects of water molecules on electrochemical kinetics, we propose a new insight into the coloring mechanism.

**Figure 4:**
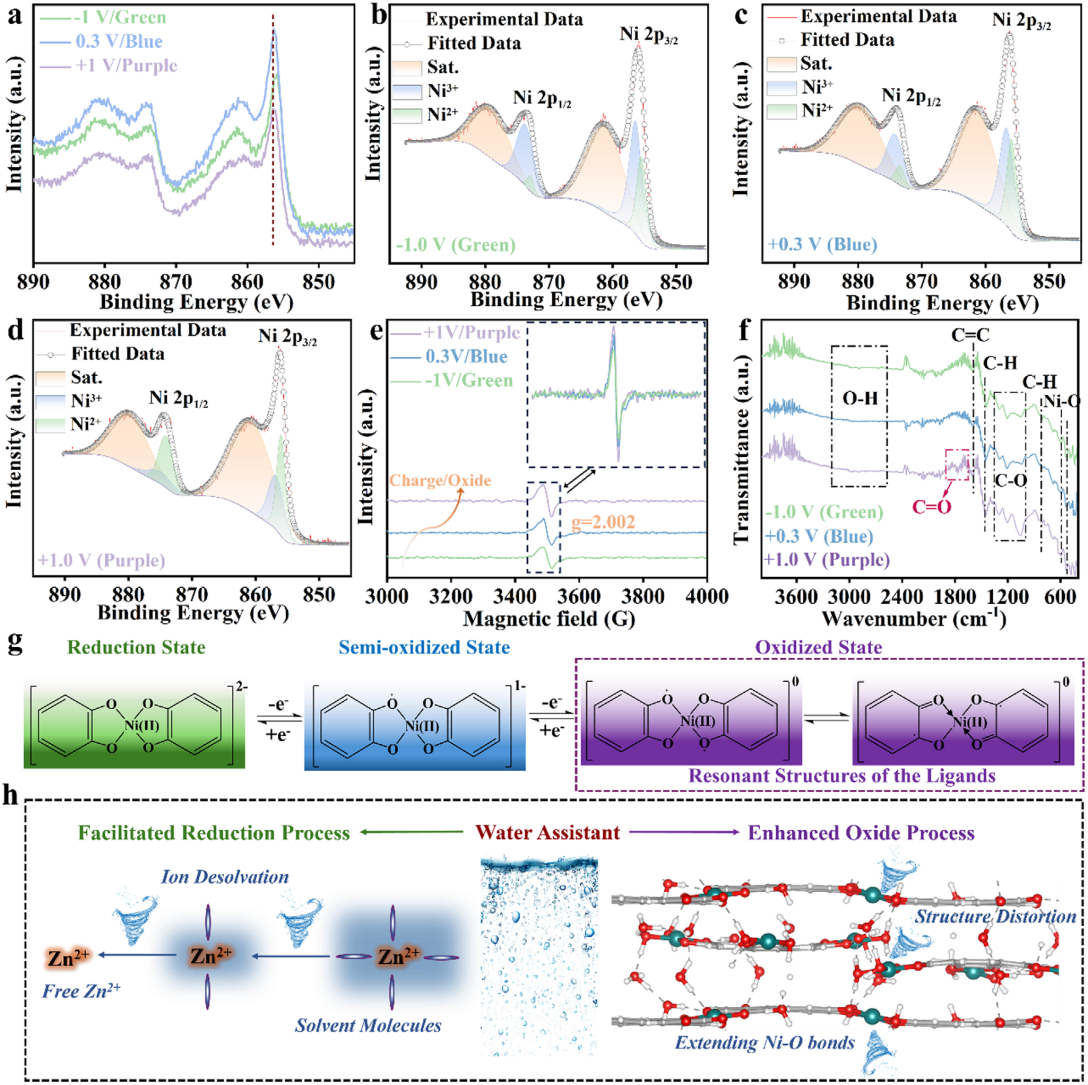
Coloring mechanism. a) Ex situ XPS survey spectra of Ni‐CAT‐1 at different voltages. XPS results for Ni 2p conducted at −1 V b), +0.3 V c) and +1 V d), respectively. e) Ex situ EPR results of Ni‐CAT‐1 at different voltages. f) Ex situ FTIR results of Ni‐CAT‐1 at different voltages. g) Schematic illustration of the coloring mechanism of Ni‐CAT‐1. h) Schematic illustration of the effects of water molecules on the coloring mechanism.

Ex situ X‐ray photoelectron spectroscopy (XPS) analysis reveals that the overall configurations of Ni 2p peaks are almost identical under different voltages, indicating that the valence of Ni has not been altered essentially (**Figure** **4**a). Achieving the ideal valence of Ni in Ni‐CAT‐1 is challenging due to the presence of rich chelating groups, multiple redox states of HHTP, impurities, and humidity, which hinder precise coordination.^[^ ^34^ ^]^ However, it is noted that the ratio of Ni 2p_1/2_ to Ni 2p_3/2_ gradually increases with the applied voltage ranging from −1 to +0.3 V, further to +1 V (Figure 4b–d). This phenomenon can well be attributed to minor variations in the ligand field surrounding the Ni atoms, potentially reflecting geometrical distortions at the Ni sites.^[^ ^14a^ ^]^ Ex situ electron paramagnetic resonance (EPR) measurements also confirm this finding. The intensity of Ni‐CAT‐1, observed at g = 2.002, exhibits an augmentation with the oxidation process, indicative of an elevated concentration of unpaired electrons surrounding the central Ni ions (Figure 4e). Besides, the gradually strengthened EPR signal also indicates the further oxidation of C─O bonds into either C─O^•^ radicals or C = O bonds.^[^ ^5^, ^35^ ^]^ In addition, Ex situ FTIR can provide further insights into the dynamic evolution of chemical bonds during the redox process. After being fully oxidized to +1 V, a new strong vibration absorption emerges ≈1750 cm^−1^, corresponding to the C = O stretching (Figure 4f). Considering the coordination numbers of oxygen and nickel, the formation of C = O bonds suggests that the interactions in the original Ni─O bonds would be weakened. This further elucidates the rationale behind the alteration in the chemical coordination environment of Ni, aligning with the findings from the XPS and EPR results. Overall, the color transitions from green (−1 V) to blue (+0.3 V) to purple (+1 V) correspond to the process of electron loss and radical generation. The specific structures in different color states, as we propose, are shown in Figure 4g. Notably, based on our DFT calculations, the introduction of water molecules can extend the bond length of Ni─O and induce structure distortions, particularly around Ni─O sites. This facilitates the oxidation coloring process by reducing the interaction of Ni─O bonds and making the formation of C = O bonds more favorable. Simultaneously, water molecules decrease the desolvation energy of Zn‐PC solvation complex structures and further accelerate Zn^2+^ diffusion, thereby promoting the reduction coloring process (Figure 4h). Therefore, water molecules do not directly contribute to this color change while indirectly act as an assistant during the whole redox process. Water molecules typically either connect with one or more Ni‐O (Ni‐O_x_) units or interact with oxygen sites within the MOF through hydrogen bonding,^[^ ^36^ ^]^ suggesting that water molecules can be stabilized within Ni‐CAT‐1 framework. Previous work demonstrated that variations in water molecules can destabilize the metal‐O units, eventually leading to the collapse of the MOF structure, which accounts for the poor cycling stability observed in aqueous electrolytes.^[^ ^36^, ^37^ ^]^ However, long‐term cycling tests in non‐aqueous electrolytes demonstrate that water molecules cannot be removed even after repeated cycles, owing to their strong hydrogen bonding within the Ni‐CAT‐1 framework.

### 2.5 Potential Applications

**Figure 5:**
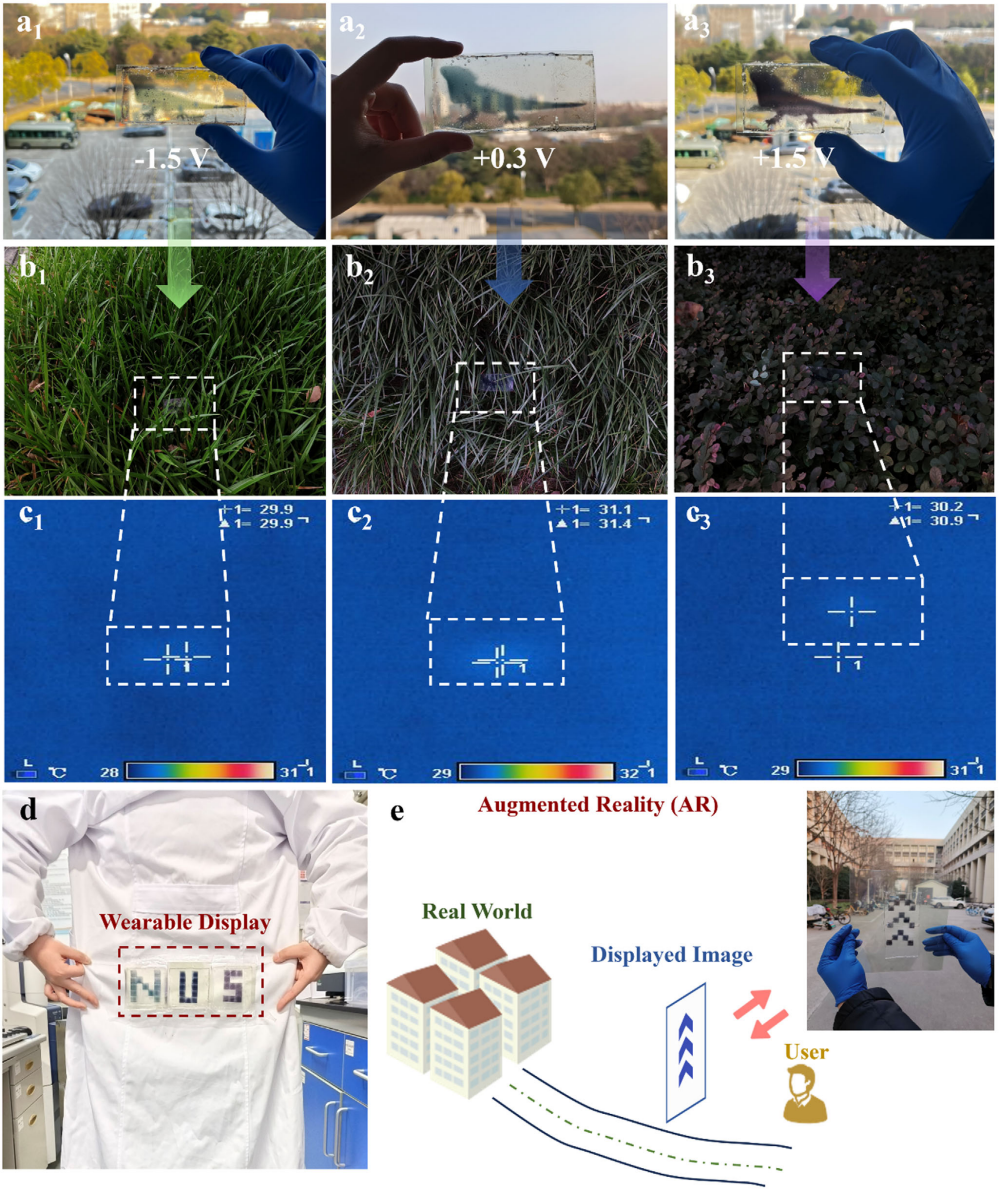
Proof‐of‐concept demonstrations of application potentials. a–c) Chameleon‐like FMEDs utilizing Ni‐CAT‐1 for active camouflage applications. a_1_–a_3_) Photographs of our FMEDs showing different colors at −1.5, +0.3, and +1.5 V. b_1_–b_3_) Photographs of FMEDs camouflaging against various backgrounds. c_1_–c_3_) Corresponding infrared thermal images of FMEDs in (b_1_‐b_3_) demonstrating infrared camouflage at high temperatures (>30 °C). d) Photograph of FMEDs in different colors attached to a human for wearable display. e) Potential applications as a handheld display for augmented reality (AR) interactions. Insert: our large‐sized FMED display.

As a proof‐of‐concept demonstration, we have developed FMEDs of various sizes for multiple applications, including adaptive camouflage (11 × 8 cm, **Figure** **5**a), flexible display (each: 6.4 × 6.4 cm, 5 × 5 pixels, Figure 5d), and augmented reality (AR) devices (20 × 30 cm, Figure 5e). All devices are assembled using PET/ITO/Ni‐CAT‐1 as the working electrodes and a zinc polyacrylamide hydrogel as the electrolyte.

Inspired by chameleons, the adaptive camouflage FMEDs are designed to exhibit vivid color‐switching capabilities, allowing them to effectively blend into their surroundings. By selectively applying voltages, such as −1.5 V for green, +0.3 V for blue, and +1.5 V for purple, the transmittance spectra from 380 to 800 nm (shown in Figure S25, Supporting Information) demonstrate excellent optical modulation capabilities. FMEDs can effectively mimic the diverse colors of plants for effective concealment (Figure 5b). Also, this chameleon‐FMED exhibits an effective infrared camouflage capability. In natural outdoor environments, typical surrounding elements such as leaves possess a high emissivity (*ɛ)* of 0.93 ranging from 2.5 to 20 µm. In contrast, flexible substrates like PET/ITO have significantly lower emissivity (*ɛ*∼0.46). This pronounced difference in radiation capability can lead to the unintended visibility of electronic devices, thereby compromising their stealth. Our chameleon‐FMED consistently maintains high emissivity across various applied voltages (*ɛ*∼0.89, 0.87, and 0.90 at −1.5, +0.3, and +1.5 V, respectively), closely matching the emissivity of surrounding leaves (Figure S26, Supporting Information). This characteristic significantly reduces infrared detectability and minimizes the temperature differential between the external environment and the chameleon‐FMED, thereby achieving all‐weather infrared camouflage, whether the external temperature is high (>30 °C) or low (<10 °C) (Figure 5c; Figure S27, Supporting Information). Future efforts shall focus on integrating this camouflage functionality into textiles, gradually developing multifunctional intelligence wearables with thermal management and flame‐retardant properties.^[^ ^38^ ^]^ Moreover, a display measuring 40.96 cm^2^ with 25 pixels, each with a size of 1 cm^2^, is fabricated (Figure 5d). This design enables stable and independent control of each pixel, achieving a dynamic display function. For instance, when voltages are set at −1.5 V, a green “N” character appears; at +0.3 V, a blue “U” character appears; and at +1.5 V, a purple “S” character appears. Thus, the letter pattern “NUS” is vividly displayed. While our previous study demonstrated that the device remained functional when immersed in water,^[^ ^25^ ^]^ repeated washing may cause displacement of the electrode layers or electrolyte leakage, potentially compromising performance. Future efforts will focus on optimizing encapsulation techniques to enhance mechanical robustness and water resistance, ensuring reliable operation under real‐world wearable conditions. In addition, owing to the transparent substrate and tunable optical modulation, the display can be used in augmented reality (AR) devices, allowing the observer to view the displayed patterns and the surrounding outdoor environment simultaneously (Figure 5e). For example, an “arrow” pattern can be displayed for road navigation (Figure S28, Supporting Information). The displayed patterns can be maintained for more than 12 h without any power input, demonstrating excellent memory performance and stability (Figure S29, Supporting Information). This new feature is particularly suitable for display applications where a high refresh rate is not consistently required.

## 3 Conclusion

In conclusion, we have developed Ni‐CAT‐1, a novel material exhibiting multicolor transitions, whereas most state‐of‐the‐art MOF systems typically display only two colors. For this unique phenomenon, we propose a new coloring mechanism based on the interconversion of C─O, C─O,^•^ and C = O, corresponding to the multicolor transitions from green to blue and then to purple. This redox process is attributed to water molecules within the MOF internal structure, which effectively enhance the switching speed (3.6/4.2 vs 4.6/6.6 s) and improve the cycling stability (2000 vs 20 cycles) compared to dehydrated samples. The underlying reasons lie in the dynamic behaviors of the metal‐oxygen bonds and ion diffusion in the color‐switching process. Water molecules facilitate the oxidation coloring process by inducing distortions in Ni‐O bonds and simultaneously aiding in the dissociation of ions from solvation complex structures to accelerate the reduction coloring process. The effect of polar molecules other than water on electrochemical behavior depends on their hydrogen bonding ability, dipole moment, molecular size, and interactions with the MOF framework. Polar protic molecules, such as alcohols (e.g., methanol and ethanol), can modify ion solvation and transport by forming hydrogen bonds with electrolyte species, potentially altering ionic mobility and charge storage capacity. Strongly polar aprotic solvents, such as dimethyl sulfoxide (DMSO) and acetonitrile, may enhance ion solvation and increase electrolyte conductivity, which can influence redox kinetics. Additionally, larger polar molecules, such as N‐methyl‐2‐pyrrolidone (NMP), may sterically hinder ion diffusion by partially blocking MOF pores, reducing electrochemical performance. Conversely, smaller polar molecules can improve electrolyte‐MOF interactions, enhancing charge transfer efficiency. These considerations suggest that the choice of polar molecules plays a critical role in tuning the electrochemical properties of MOFs, and further investigations are clearly needed to systematically explore these effects. In addition, future research may focus on selecting appropriate metal ions and exploring alternative organic ligands with multiple redox states, as well as investigating their dynamic interactions under external electric fields.

## Conflict of Interest

The authors declare no conflict of interest.

## Author Contributions

Q.Z. and J.Y. contributed equally to this work. Q. Z, Y. F. G, Y. W. Z, and J. W designed and conceptualized the study. Q. Z, W. W, Y. L. G, and X.C completed the experimental section. J.Y. and H.Y. performed the DFT calculations. X.Y.W conducted cryo‐TEM tests. Q.Z, J.Y, Y.F.G, Y.W.Z, and J.W wrote the manuscript and supervised the projects. All authors reviewed and approved the manuscript.

## Supporting information



Supporting Information

Supplemental Video 1

## Data Availability

The data that support the findings of this study are available from the corresponding author upon reasonable request.
