# Supplementary material for: Water‐Enhanced Multicolor Electrochromism in Nickel‐Catecholate MOFs
Source: Adv Sci (Weinh). 2025 Mar 27;12(25):2500678. doi: 10.1002/advs.202500678 (PMC12224994; doi:10.1002/advs.202500678)
Supplement: Supplementary file 1 — Supporting Information [file ADVS-12-2500678-s001.docx]

***Supporting Information***

**Water-enhanced Multicolor Electrochromism in Nickel-catecholate MOFs**

*Qi Zhao,^1#^ Jing Yang,^2#^ Xingyang Wang,^1^ Wanwan Wang,^5^ Yulin Gao,^1^ Xue Chen,^3^ Jianguo Sun,^1^ Hao Yuan,^2^ Yu Liu,^1*^ Jinwoo Park,^4^ Lewis Kien Juen Ting,^1^Qing Wang,^1^ Pooi See Lee,^4^ Yanfeng Gao,^3,7*^ Yong-Wei Zhang,^2*^ John Wang^1,6*^*

^1^ Department of Materials Science and Engineering, National University of Singapore, Singapore 117575, Singapore

^2^ Institute of High Performance Computing (IHPC), Agency for Science, Technology and Research(A*STAR), 1 Fusionopolis Way, #16-16 Connexis, Singapore 138632, Republic of Singapore

^3^School of Materials Science and Engineering, Shanghai University, Shanghai 200444, P. R. China

^4^ School of Materials Science and Engineering, Nanyang Technological University, 50 Nanyang Avenue, Singapore 639798, Singapore

^5^Institute of Materials Research and Engineering (IMRE), Agency for Science, Technology and Research (A*STAR), 2 Fusionopolis Way, Innovis #08-03, Singapore 138634, Republic of Singapore

^6^ National University of Singapore (Chongqing) Research Institute, Chongqing Liang Jiang New Area, Chongqing 401120, China

^7^School of Chemical and Environmental Engineering, Anhui Polytechnic University, Wuhu 241000, P. R. China

^#^These authors contributed equally to this work

*Corresponding author

**1 Experimental procedures and simulation details**

**1.1 Materials**

Nickel(II) acetate tetrahydrate (Ni(OCOCH_3_)_2_·4H_2_O, trace metals basis, ≥ 99.9%), N,N-Dimethylformamide (DMF, ACS reagent, ≥ 99.8%), chloroform (CHCl_3_, anhydrous, ≥ 99%), reagent alcohol (anhydrous, ≥ 99.5%), zinc perchlorate hexahydrate, propylene carbonate (anhydrous, 99.7%) were purchased from Sigma-Aldrich. 2,3,6,7,10,11-hexahydroxytriphenylene hydrate (HHTP) was purchased from Tee Hai Chem Pte. Ltd. (Singapore). Acrylamide (AM; 99%), a-ketoglutaric acid (AK; 99%), ammonium persulphate (APS; 99%), and N, N’-methylenebisacrylamide (MBAA; 99%) were purchased from Aladdin. The deionized (DI) water (>18 MΩ) was produced in the laboratory. Indium tin oxide (ITO)-coated PET (1.0 mm thickness, 20 Ω sheet resistance) was purchased from Zhuhai Kaivo Optoelectronic Technology Co. Ltd. (China). All materials were used without further purification.

**1.2 Synthesis of Ni-CAT-1 and De-Ni-CAT-A**

For the synthesis of Ni-CAT-1, firstly, 0.2 g of nickel(II) acetate tetrahydrate (Ni(OCOCH_3_)_2_·4H_2_O) was dissolved in 20.0 mL DI water and 0.13 g of 2,3,6,7,10,11-Hexahydroxytriphenylene (HHTP) was dissolved in 20.0 mL DMF, respectively. Next, the above two were both dissolved into 40.0 mL DI water under sonication and magnetic stirring for 30 min to ensure mixing evenly. The reaction mixture was heated in an isothermal oven at 95 °C for 12 h, resulting in small dark blue crystals. The mixture was then allowed to cool naturally to room temperature and the crystals were washed with DI water, and then ethanol (repeat several times, respectively). After decanting, the remaining powder was dried at 60 °C for 24 hours to form Ni-CAT MOFs.

For the synthesis of De-Ni-CAT-1, the previously obtained Ni-CAT-1 powder was dissolved into 20 mL chloroform under magnetic stirring for 12 h. Following, the mixed solution was heated at 50 °C under vacuum. Finally, the resulting De-Ni-CAT-1 powder was stored in a glove box.

**1.3 Solution process fabrication of the Ni-CAT-1 and De-Ni-CAT-1 electrode**

Ni-CAT MOFs dispersions were made by mixing Ni-CAT MOFs powders (10 mg) and alcohol (1 ml). Then, an airbrush was used to spray the as-prepared dispersions onto an ITO-PET. The distance between the nozzle and the post-treated ITO glass was maintained at 15 cm. The nozzle diameter was 0.3 mm, and the spraying pressure was 0.1 MPa. Finally, all as-prepared films were annealed at 80 °C for 30 min.

**1.4 Synthesis of zinc polyacrylamide hydrogel electrolyte**

Zinc polyacrylamide hydrogel electrolyte was prepared based on our previous work. Typically, Zn(ClO_4_)_2_ was added to a 2.2 M AM (monomer) solution containing 0.05 mol % of MBAA as a cross-linking agent and 1 mol% of APS as a catalyst, along with 0.1 mol % of AK as a photoinitiator. Subsequently, the solution was mixed using a vortex mixer for 10 mins to achieve a homogeneous transparent solution. Following this, the precursor solution was degassed with N_2_ for 0.5 h. Finally, the solution was transferred into a Teflon mold and cured using the UV light treatment for 30 mins.

**1.5 Assembly of the FMEDs**

Different-sized FMEDs, including a display (length ×width: 6.4 cm × 6.4 cm, pixel: 1 cm × 1 cm), an AR device (20 cm × 30 cm), and a bioinspired chameleon-camouflaged device (11 cm ×8 cm), were assembled using the Ni-CAT-1 electrode and the zinc polyacrylamide hydrogel electrolyte.

**1.6 Characterization**

The material structure was characterized by X-ray diffraction (XRD, Rigaku Ultima Ⅳ. Fourier transform infrared (FTIR) spectra of the Ni-CAT-1 and De-Ni-CAT-1 samples were acquired on an Agilent Cary 660 FT-IR Spectrometer. Meanwhile, the ITO substrate signal was collected as background in the 400-4000 cm^-1^ range. Raman analysis (LabRAM HR Evolution spectrometer) was applied to determine bonding structures in each of the samples. Raman spectrum was calibrated by using Si sample and aligning the peak to 520.7 cm^-1^. Electron paramagnetic resonance (EPR, Bruker EMXnano) was used to confirm the concentration of unpaired electrons in different color states. X-ray photoelectron spectroscopy (XPS, Kratos AXIS Supra^+^, Kratos Analytical Ltd) was used to analyze the elemental composition and chemical state of electrodes under different voltages. The XPS spectra was calibrated by aligning C 1s peak to 284.8 eV. Cryo-Transmission electron microscopy (TEM) was conducted on a JEOL 2800 at -165 ℃, assembled with a Melbuild Double tilt LN2 Vacuum Transfer Holder. The optical transmittance of the electrode was characterized by a UV/Vis/NIR spectrophotometer (UH4150; Hitachi, Japan). Electrochemical and electrochromic measurements were carried out in a quartz cell containing 1 M Zn(ClO_4_)_2_/PC on an electrochemical workstation (CHI760E, CHI Instruments, USA) using a three-electrode system. A standard Ag/AgCl electrode and a platinum plate were used as the reference and counter electrodes, respectively. In the two-electrode system, zinc polyacrylamide hydrogel electrolyte was employed for quasi-solid-state FMEDs.

**1.7 Calculation details**

Density functional theory (DFT) calculations were carried out using the Vienna Ab initio Simulation Package (VASP).^1^ The exchange-correlation potential was analyzed using the generalized gradient approximation (GGA) in the Perdew-Burke-Ernzerhof (PBE) formulation.^2^ A cutoff energy of 500 eV was applied to the plane-wave basis set. The thresholds for force and energy convergence were set to 0.05 eV Å^−1^ and 1 × 10^−6^ eV, respectively. To balance accuracy and computational resource usage, only the Gamma point was considered during the structure optimization. Grimme’s DFT-D3 method was employed to include long-range van der Waals corrections.^3^ Ab initio molecular dynamics (AIMD) simulations were conducted in a canonical (NVT) ensemble using a Nosé thermostat at 300 K^4^ and 10 ps simulations were performed with a time step of 1 fs. To study the effect of water on the ion desolvation, the following desolvation steps were studied:

Without water:

1^st^ PC: ${Zn(PC)}_{4}^{2+}$ = ${Zn(PC)}_{3}^{2+}$+PC

2^nd^ PC: ${Zn(PC)}_{3}^{2+}$ = ${Zn(PC)}_{2}^{2+}$+PC

3^rd^ PC: ${Zn(PC)}_{2}^{2+}$ = Zn(PC)^2+^+PC

4^th^ PC: Zn(PC)^2+^=Zn^2+^+PC

With water:

1^st^ PC: ${Zn(PC)}_{4}^{2+}$ + H_2_O = Zn(PC)_3_(H_2_O)^2+^+PC

2^nd^ PC: Zn(PC)_3_(H_2_O)^2+^= Zn(PC)_2_(H_2_O)^2+^+PC

3^rd^ PC: Zn(PC)_2_(H_2_O)^2+^= Zn(PC)(H_2_O)^2+^+PC

4^th^ PC: Zn(PC) (H_2_O)^2+^= Zn(H_2_O)^2+^+PC

Zn(H_2_O)^2+^=Zn^2+^+H_2_O

**2 Supplementary Notes**

**Supplementary Note 1:**

Raman bands observed at 300-800 cm^-1^ are associated with Ni-O bonds, while those at 1300-1500 cm^-1^ corresponded to the C=C bonds (**Figure S5a**).^5,6^ The FTIR peaks observed at 1643 cm^-1^, 1454 cm^-1^ correspond to the C=C and C-H bonds, respectively, attributed to the aromatic ring **(Figure S5b)**.^7,8^ The wavenumber regions of 1300-1200 cm^-1^,850-750 cm^-1^ and 700-500 cm^-1^ are associated with C-O, C-H bending and Ni-O stretching vibration, respectively, which are typical characteristics of Ni-HHTP. ^9-11^

**Supplementary Note 2:**

De-Ni-CAT-1 also shows a nanorod-like morphology, with an almost identical size to Ni-CAT-1 (**Figure S9**). EDS mapping confirms the existence of C, O, Ni elements (**Figure S10**). Moreover, the typical (100) and (001) crystal planes of De-Ni-CAT-1 correspond to the lattice spacing of 1.96 nm and 0.32 nm, respectively (**Figure S11**).^12^ Large pores (≈2 nm) in a honeycomb arrangement can also be formed (**Figure S12**). Basically, dehydration process has a rather limited effect on morphology and crystal structure of Ni-CAT-1.

**Supplementary Note 3:**

The Ni-CAT-1 and De-Ni-CAT-1 powders were compressed at a pressure of 600 MPa into 10 mm round pellets of approximately 0.3 mm thick. Conductivity measurements were carried out on these pellets with a four-point probe instrument. The conductivity *σ* is given by Equation 1, where *t* is the pellet thickness, *V* and *I* are the measured voltage and current respectively, and *f_1_* and *f_2_* are geometric correction factors. *f_1_* is given by Equation 2, where s is the probe spacing, and *f_2_* was determined from standard correction factor tables to be 0.92 for all samples. At least 10 current measurements were taken within a voltage range of 5-120 mV, and the effective *V/I* was obtained from the line of best fit of the resultant *V/I* plot

$\sigma^{-1}=\rho=\frac{\pi}{ln(2)}t\left( \frac{V}{I} \right)f_{1}f_{2}$ (1)

$f_{1}=\frac{ln(2)}{\ln\left[ \frac{\sinh t/s}{\sinh t/{2s}} \right]}$ (2)

**Supplementary Note 4:**

The optical energy band gaps were calculated using the Kubelka–Munk function:

αhν=B(hν-E_g_ )^n^

where 𝛼 is the absorption coefficient, h𝜈 represents the photon energy (eV) (with h as the Planck constant, approximately 4.13567 × 10⁻^15^ eV·s), and 𝜈 is the frequency of the incident light (𝜈 = c/𝜆, where c is the speed of light, approximately 3 × 10^8^ m s^-1^). 𝜆 is the wavelength of the incident light (nm), B is a proportionality constant, and E_g_ denotes the bandgap. Our samples are direct bandgap semiconductors, n=1/2.

**Supplementary Note 5:**

From green (-1 V) to purple (+1 V), Ni-CAT-1 exhibits high areal capacities of 132.5, 83.2, 62.0 and 25.9 mAh m^-2^ at current densities of 0.5, 0.8, 1 and 2, respectively (**Figure S18a**). Similarly, from green (-1 V) to blue (+0.3 V), the areal capacities of 40.8, 32.0, 17.2, 14.9 and 6.6 mAh m^-2^ are achieved at current densities of 0.3, 0.5, 0.8, 1 and 2 mA cm^-2^, respectively (**Figure S18b**); from blue (+0.3 V) to purple (+1 V), the areal capacities of 85.9, 44.0, 28.8, 20.6 and 8.1 mAh m^-2^ are achieved at current densities of 0.3, 0.5, 0.8, 1 and 2 mA cm^-2^, respectively (**Figure S18c**). Comparably, De-Ni-CAT-1 shows a relative moderate energy storage capacity in different color states (**Figure S20-S21**). From green (-1 V) to blue (+0.3 V), the areal capacities of 30.8, 21.5, 11.2, 7.6 and 2.9 mAh m^-2^ are achieved at current densities of 0.3, 0.5, 0.8, 1 and 2 mA cm^-2^, respectively; from blue (+0.3 V) to purple (+1 V), the areal capacities of 44.8, 35.6, 12.8, 10.1 and 3.5 mAh m^-2^ are achieved at current densities of 0.3, 0.5, 0.8, 1 and 2 mA cm^-2^, respectively. Notably, the areal capacities of the Ni-CAT-1 are consistently higher than the that of the De-Ni-CAT-1, suggesting that water plays an important role in electrochemical kinetics.

**3 Supporting Figures**

**
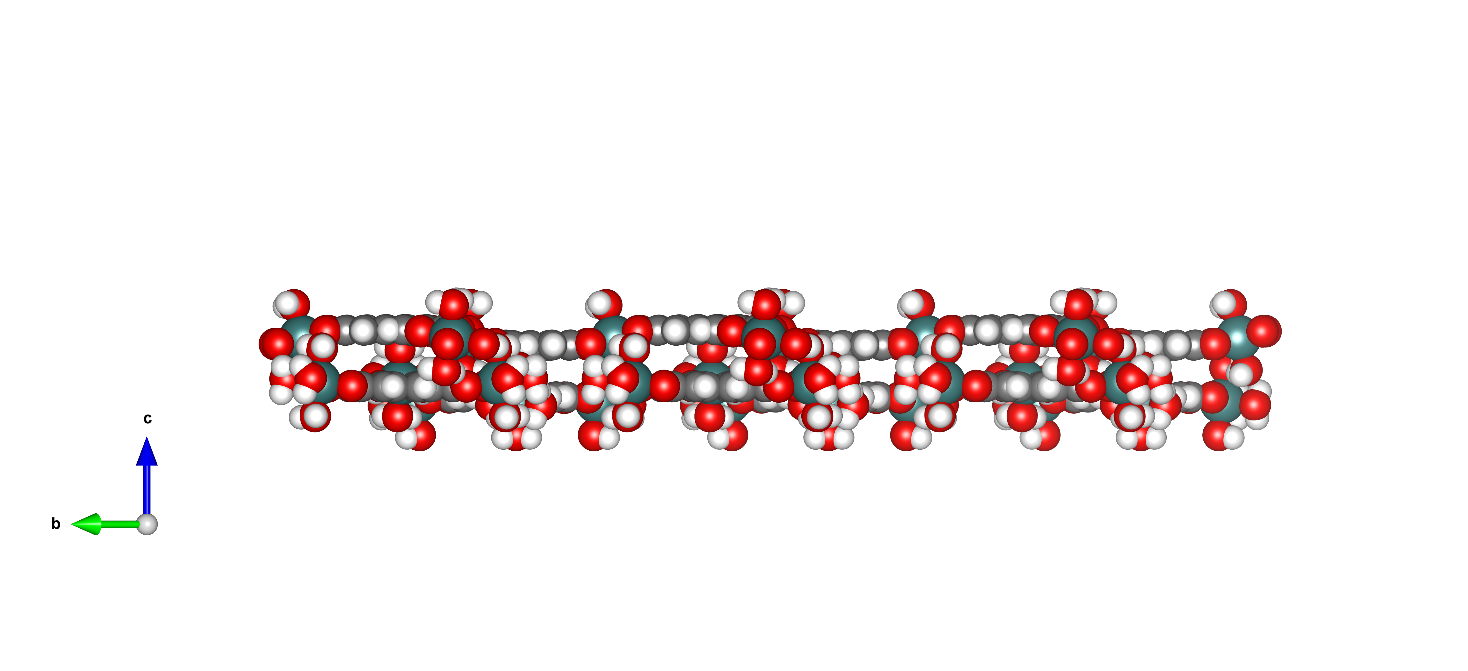
**

**Figure S1.** Structure of Ni-CAT-1 along the [110] direction. Color indicators: green, Ni; red: O; gray: C; white: H.

**
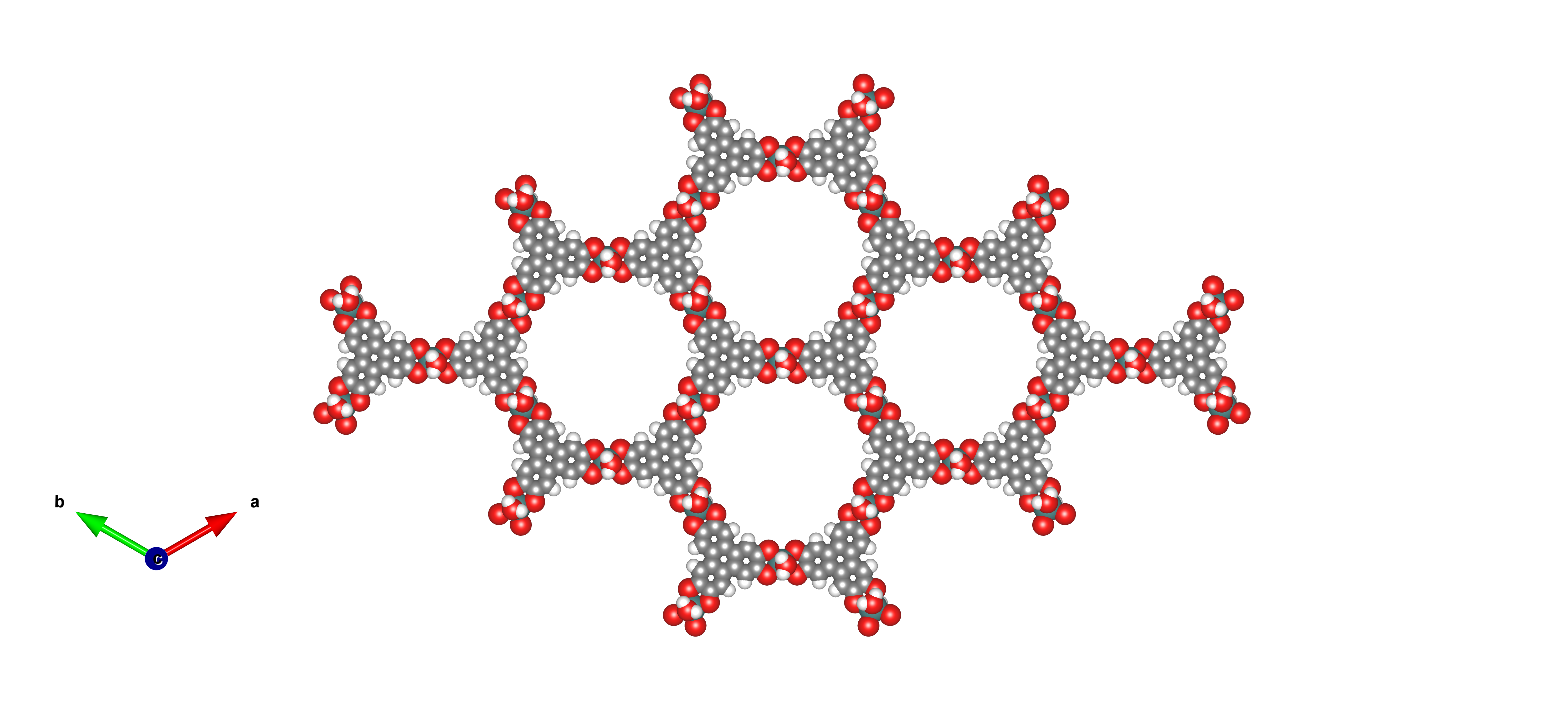
**

**Figure S2.** One layer of Ni-CAT-1 consists of extended honeycomb-like structures. Color indicators: green, Ni; red: O; gray: C; white: H.


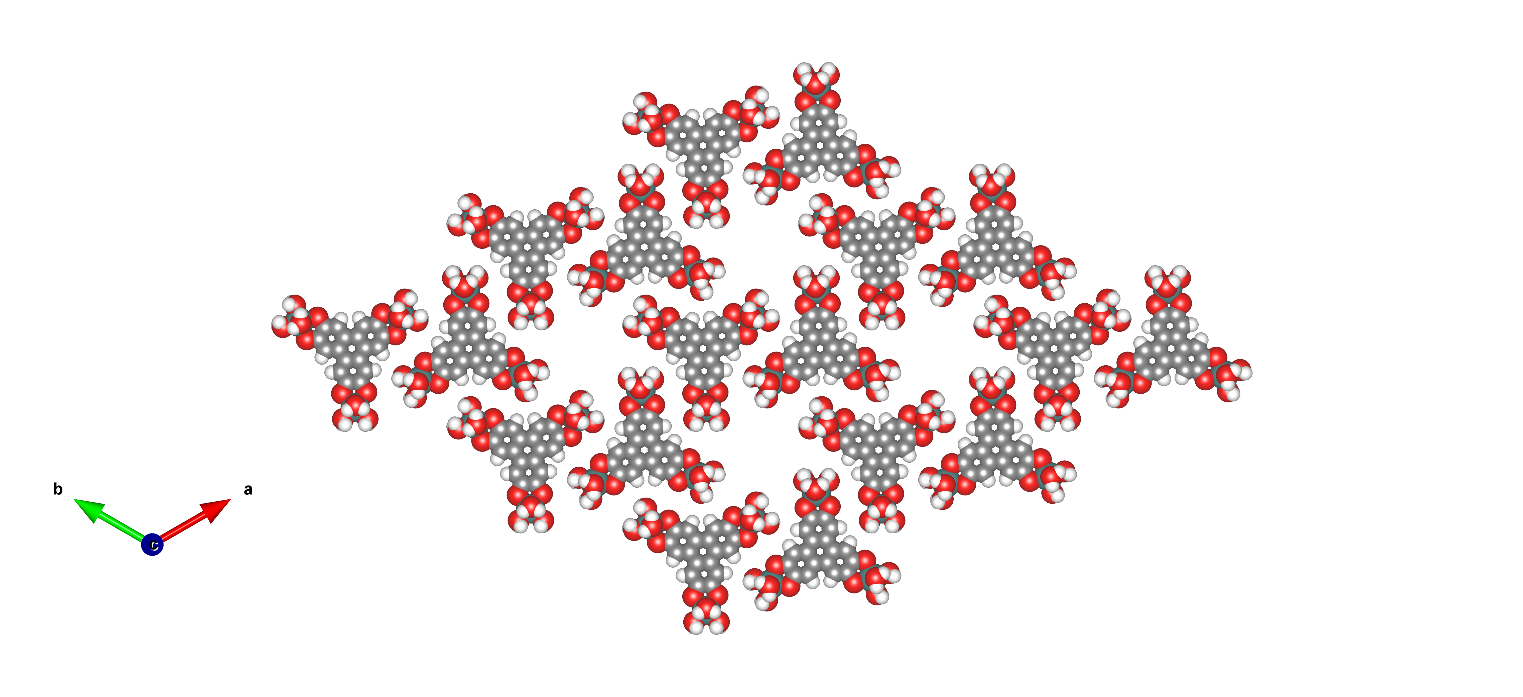


**Figure S3.** The other layer of Ni-CAT-1 consists of discrete units. Color indicators: green, Ni; red: O; gray: C; white: H.


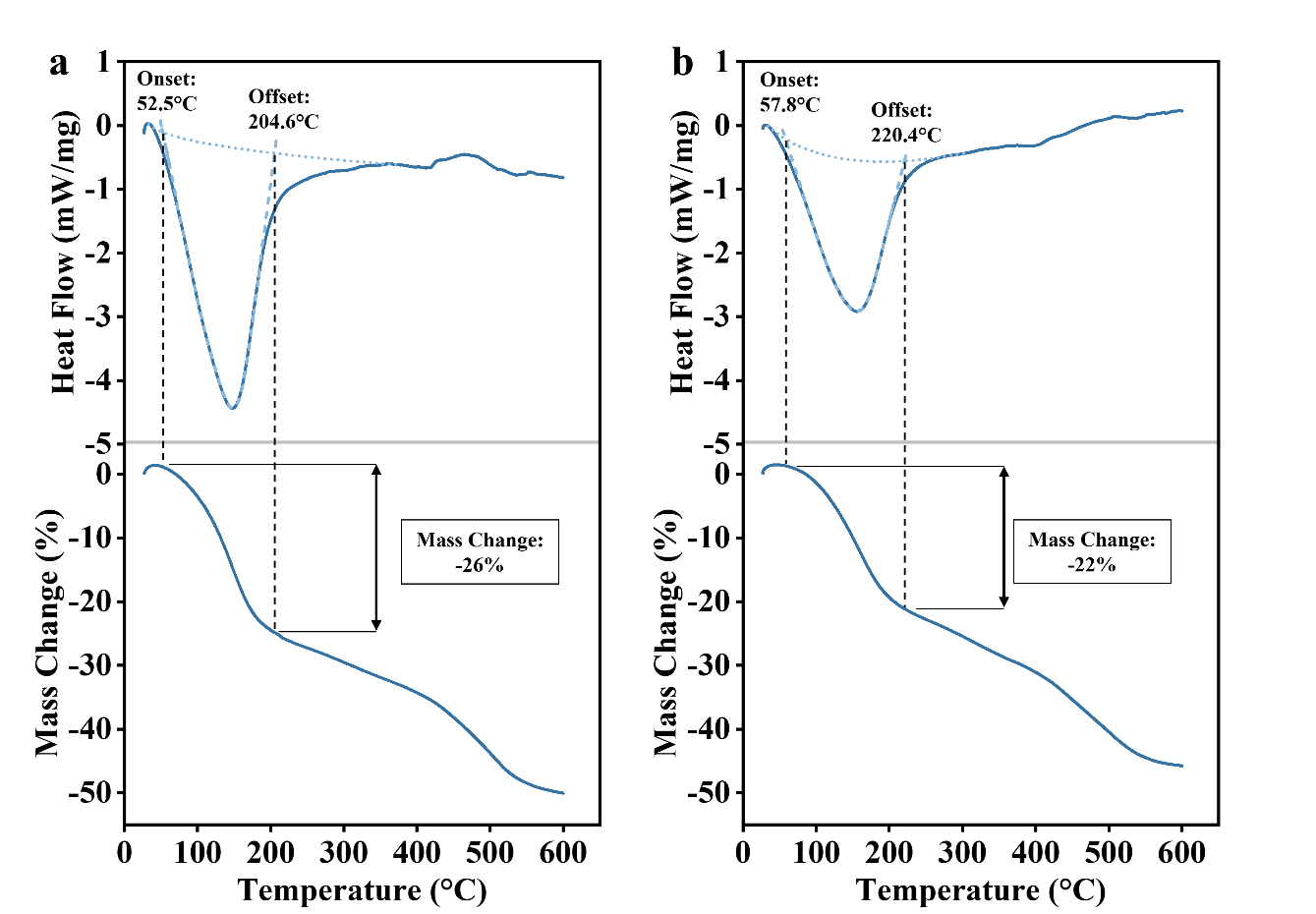


**Figure S4.** Simultaneous DSC and TG analysis of (a) Ni-CAT-1 and (b) De-Ni-CAT-1.


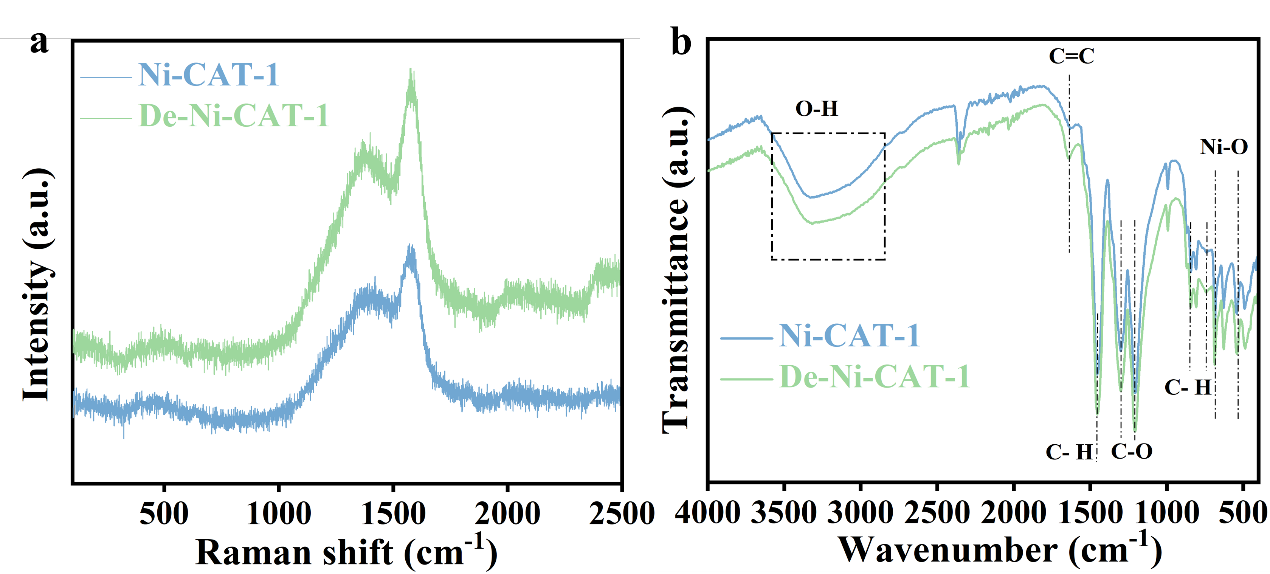


**Figure S5.** Raman (a) and FTIR (b) results of Ni-CAT-1 and De-Ni-CAT-1.


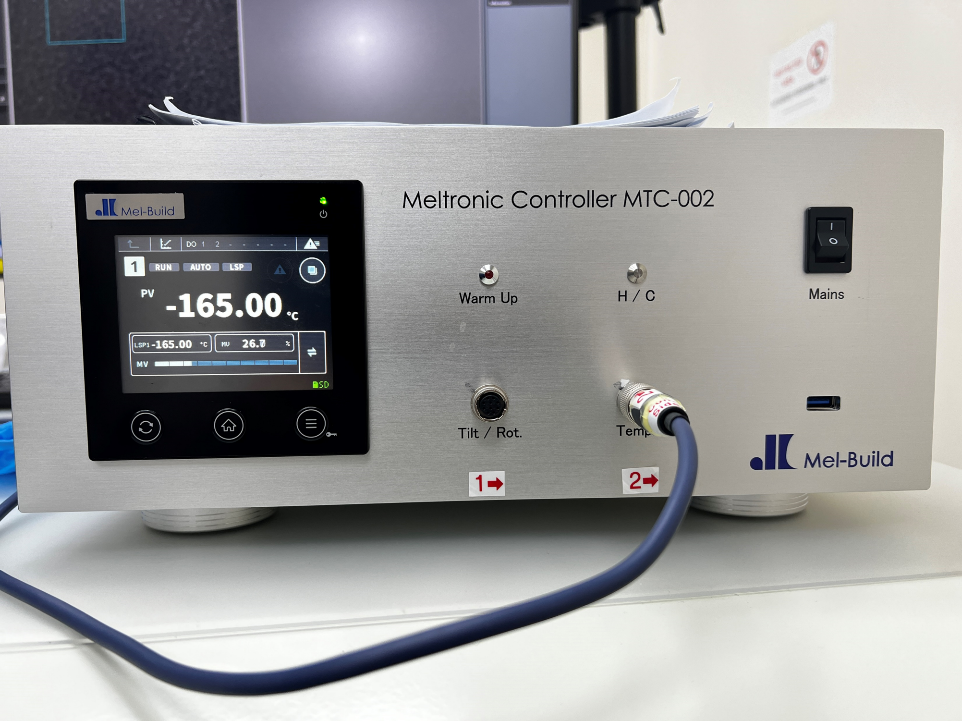


**Figure S6.** Cryo-TEM test conducted at -165 °C.


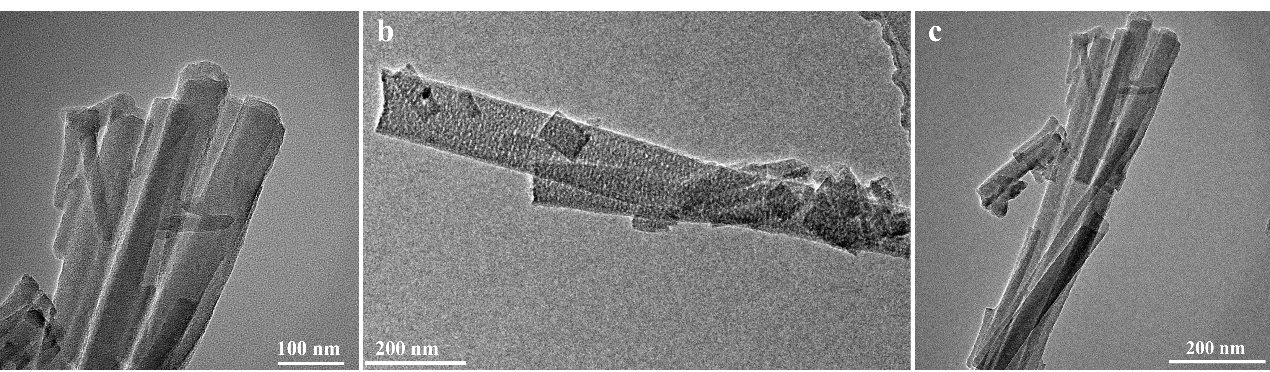


**Figure S7.** Morphology of Ni-CAT-1.


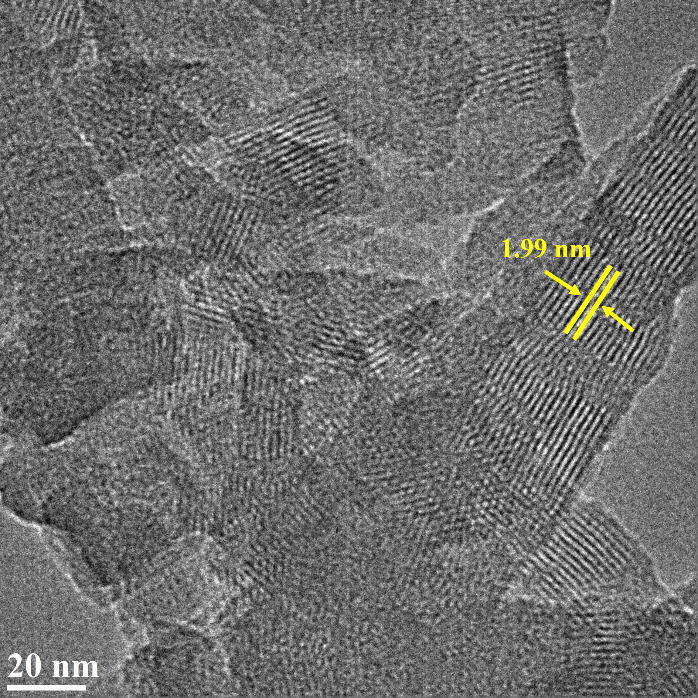


**Figure S8.** Lattice stripes of Ni-CAT-1.


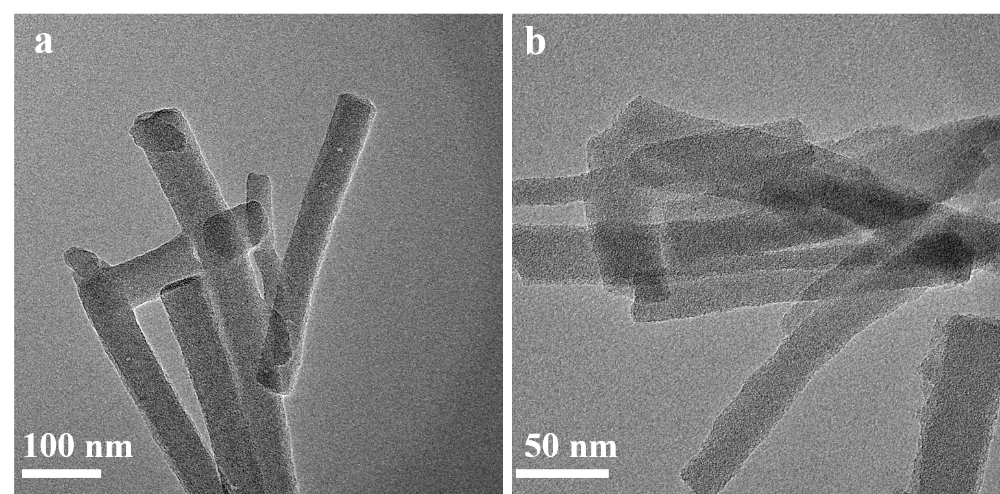


**Figure S9.** Morphology of De-Ni-CAT-1.

**
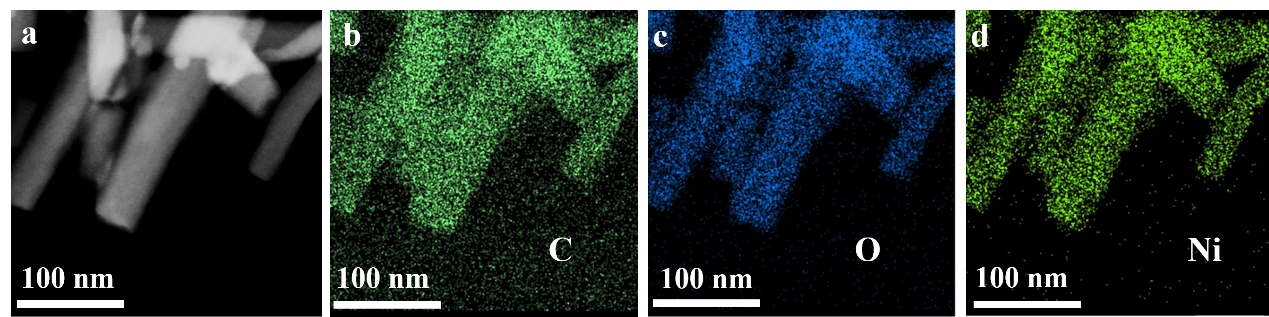
**

**Figure S10.** EDS elemental mapping results of De-Ni-CAT-1.


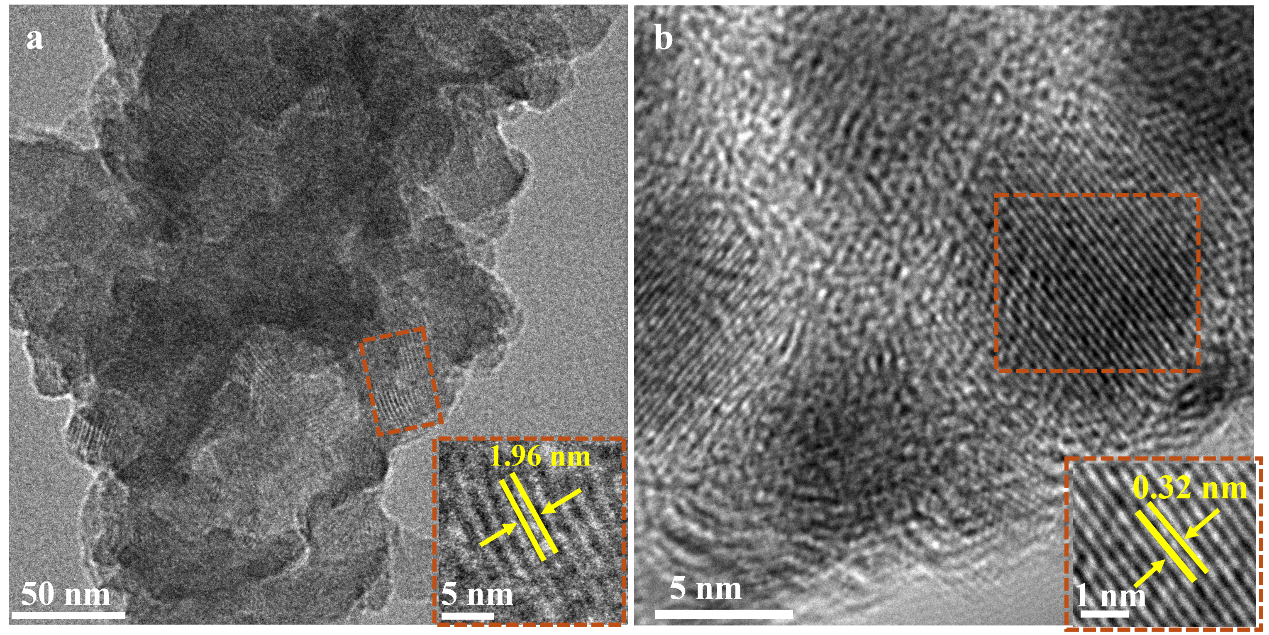


**Figure S11.** Lattice stripes of De-Ni-CAT-1.

**
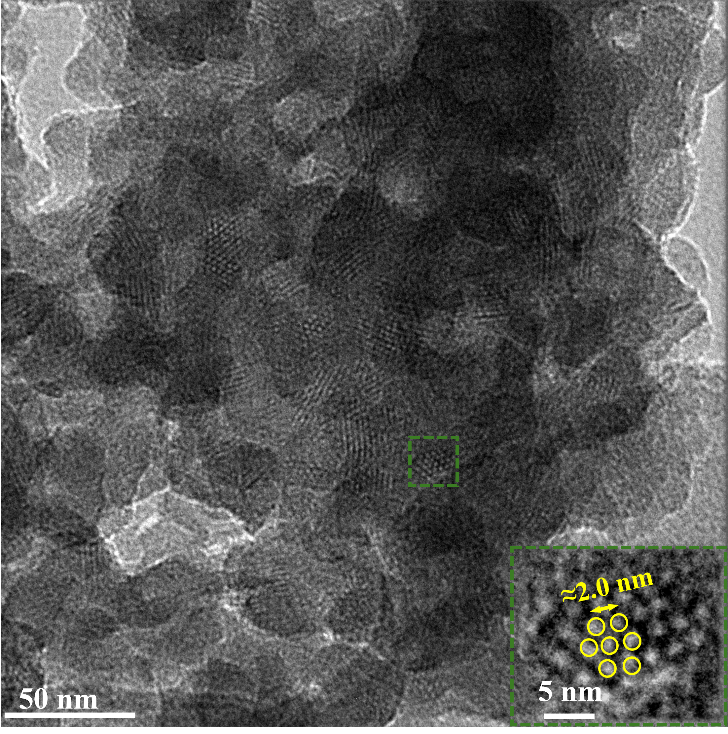
**

**Figure S12.** Cryo-TEM image of De-Ni-CAT-1 enlarged from the selected green area along [001] that shows a hexagonal pore packing with d100 ≈ 2.0 nm.

**
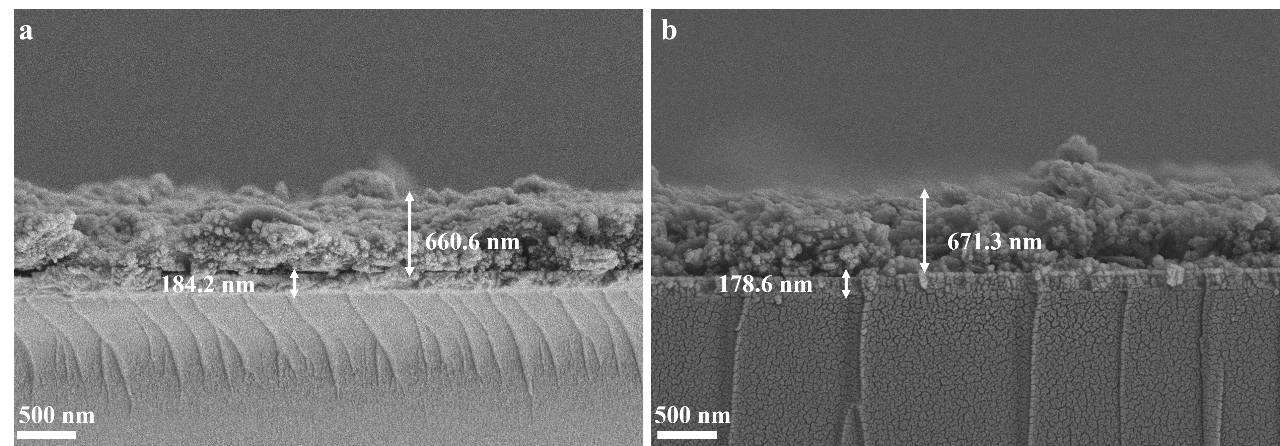
**

**Figure S13.** Cross-section SEM image of the (a) Ni-CAT-1 and the (b) De-Ni-CAT-1. The thicknesses of Ni-CAT-1 and De-Ni-CAT-1 are 660.6 nm and 671.3 nm, respectively. The thickness of ITO/PET is around 180 nm.


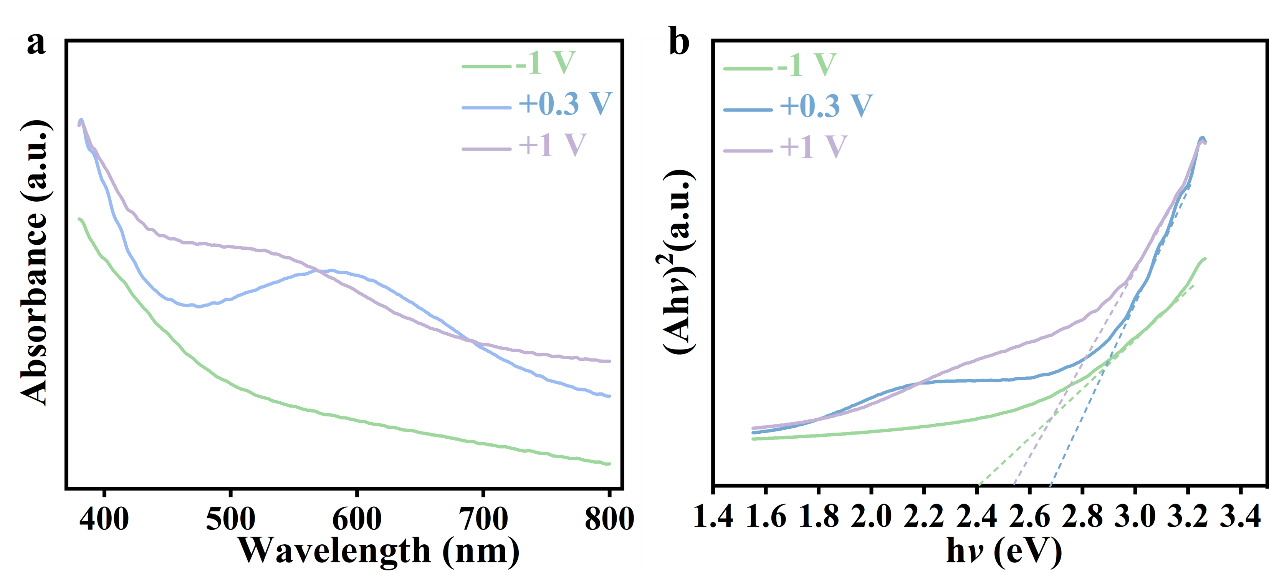


**Figure S14.** (a) Absorbance spectra for Ni-CAT-1 (b) Tauc plots of (αh*ν*)^2^ versus h*ν* for Ni-CAT-1 at potentials of -1 V, +0.3 V and +1V, respectively.


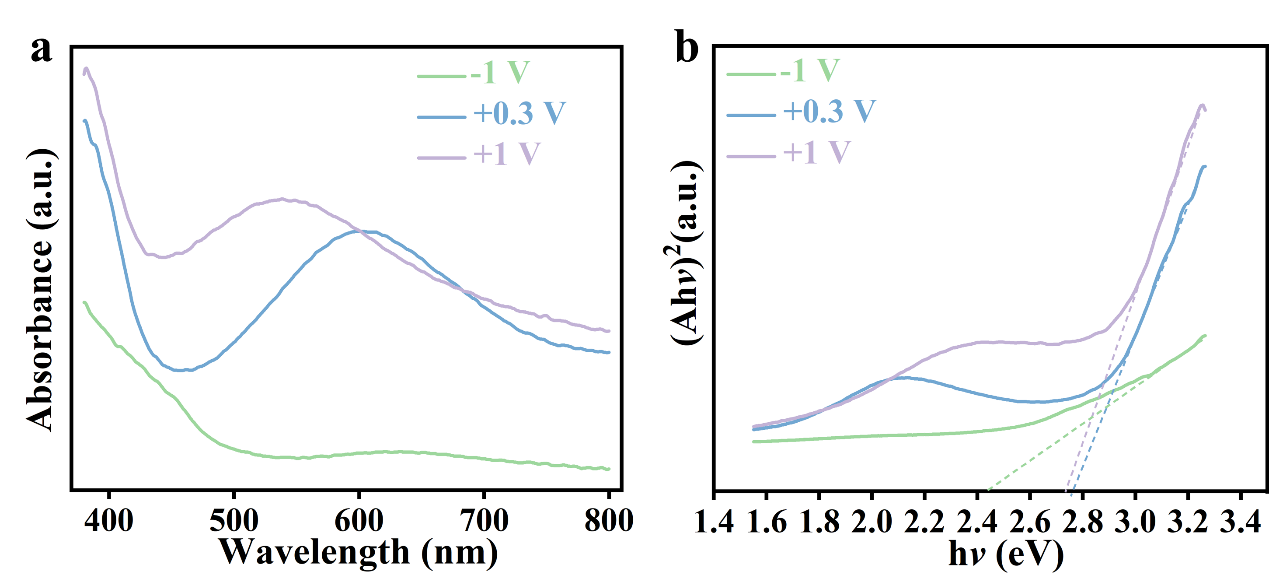


**Figure S15.** (a) Absorbance spectra for De-Ni-CAT-1 (b) Tauc plots of (αh*ν*)^2^ versus h*ν* for De-Ni-CAT-1 at potentials of -1 V, +0.3 V and +1V, respectively.

**
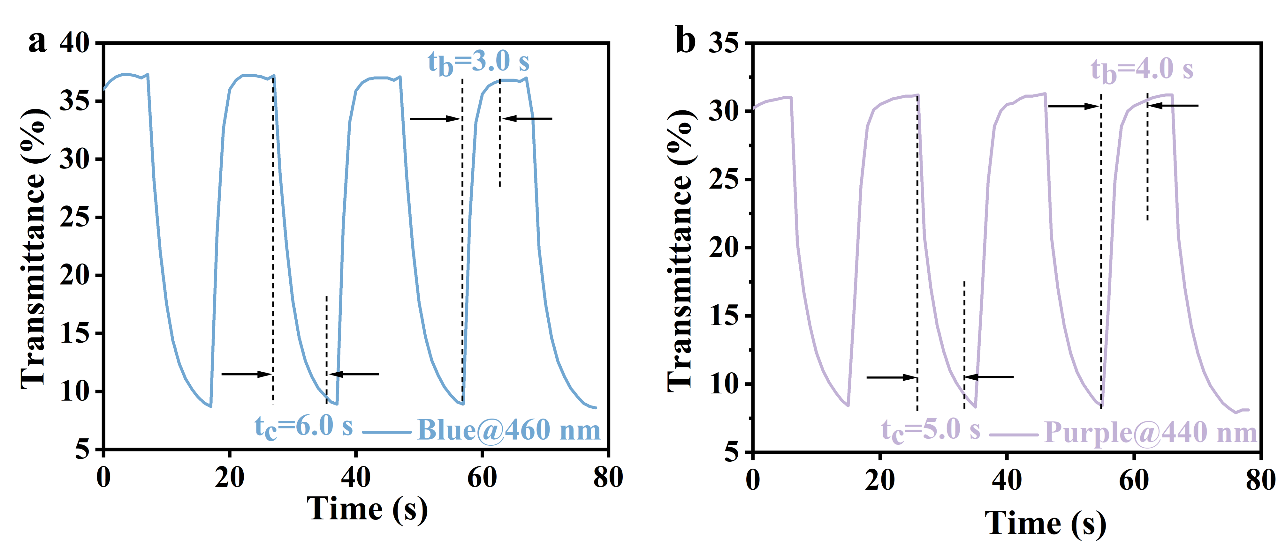
**

**Figure S16.** Switching speeds of Ni-CAT-1 at (a) 460 nm and (b) 440 nm.

**
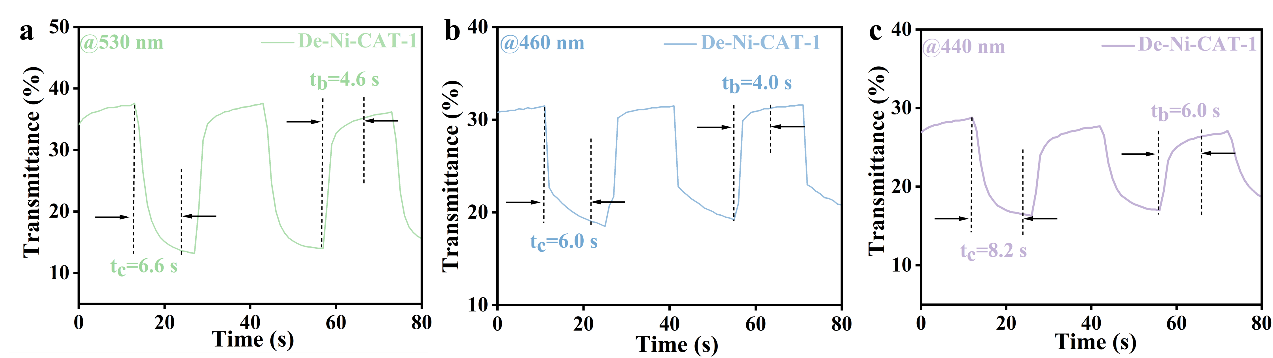
**

**Figure S17.** Switching speeds of De-Ni-CAT-1 at (a) 530 nm, (b) 460 nm and (c) 440 nm.

**
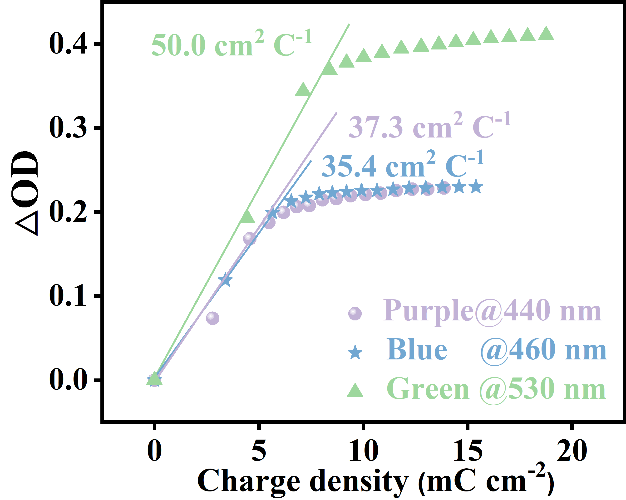
**

**Figure S18.** Coloration efficiency of De-Ni-CAT-1 at different wavelengths.


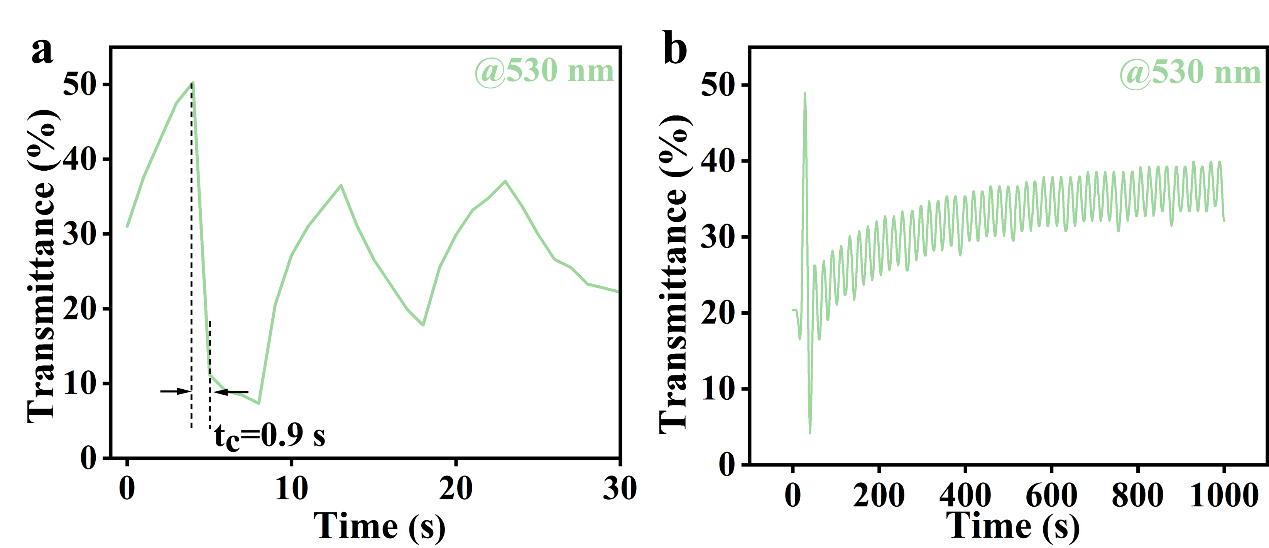


**Figure S19. Electrochromic performance of Ni-CAT-1 in an aqueous electrolyte.** (a) Switching speed and (b) cycling life. Aqueous electrolyte is prepared using 1 M ZnCl_2_ in deionized water.

**
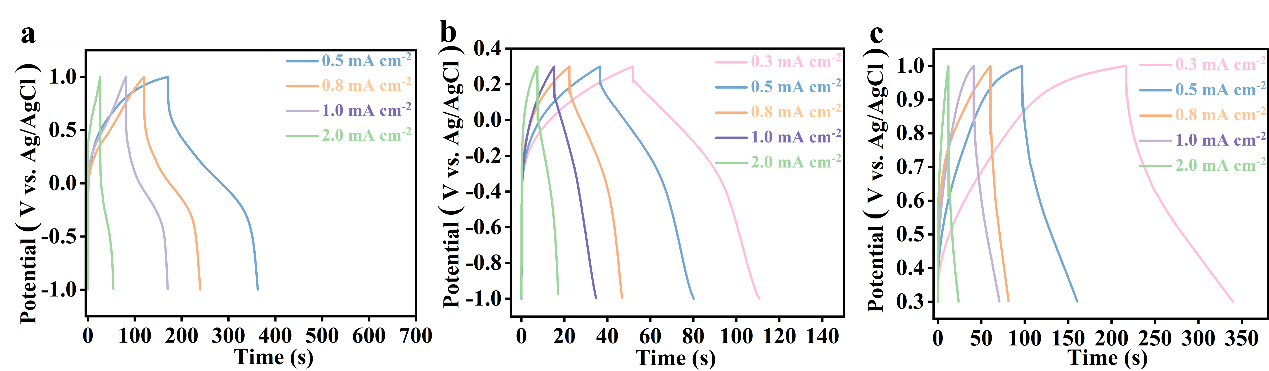
**

**Figure S20**. Galvanostatic charge/discharge curves of Ni-CAT-1 at different current densities.

**
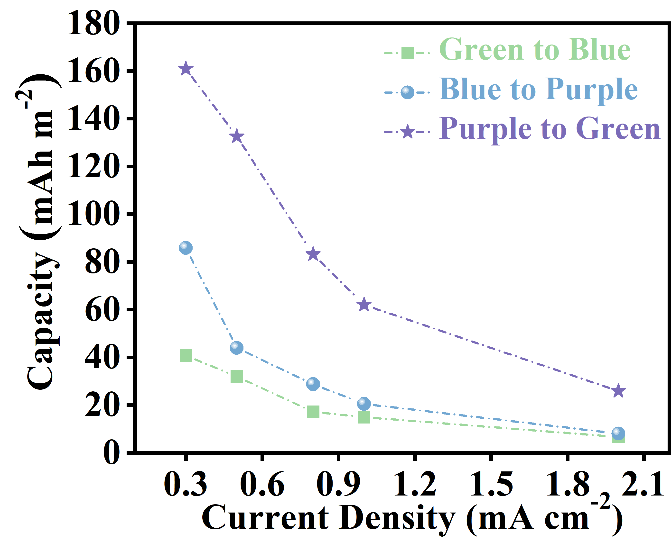
**

**Figure S21**. Summary of areal capacities of Ni-CAT-1 in different colors.

**
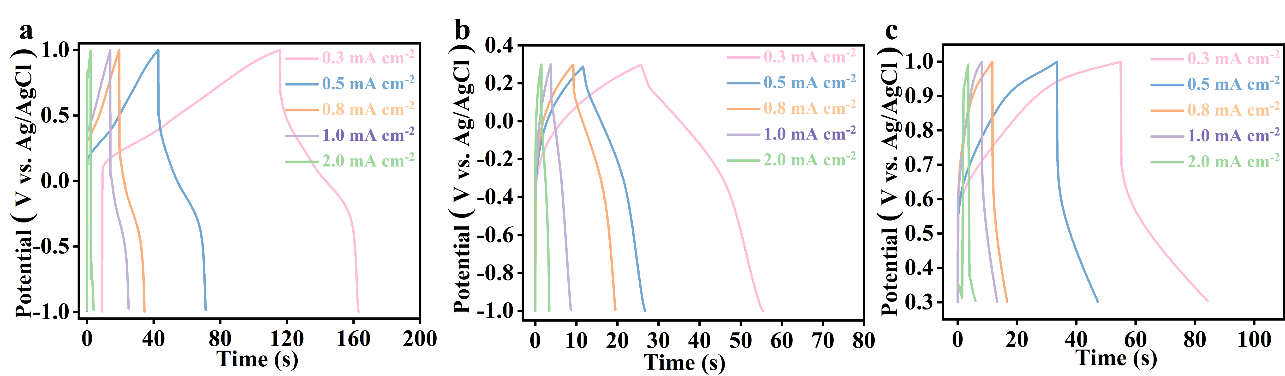
**

**Figure S22**. Galvanostatic charge/discharge curves of De-Ni-CAT-1 at different current densities.

**
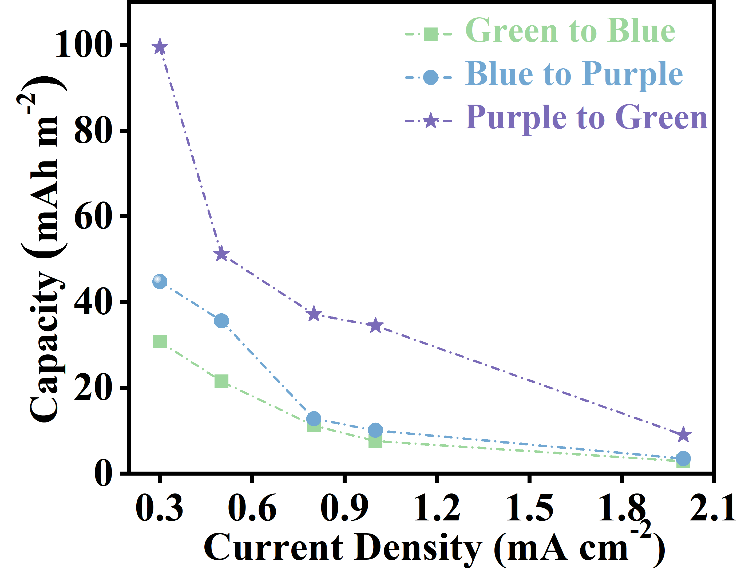
**

**Figure S23**. Summary of areal capacities of De-Ni-CAT-1 in different colors.


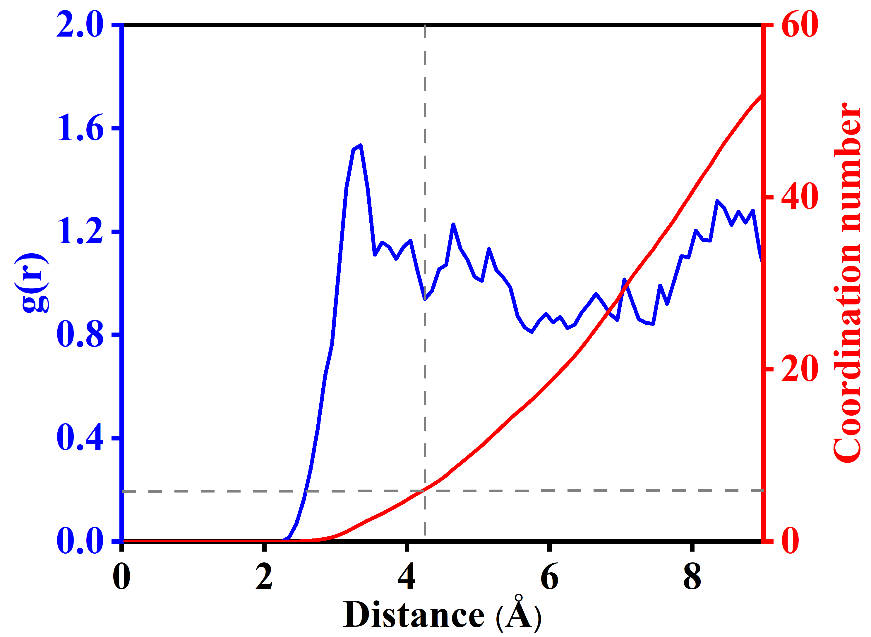


**Figure S24**. Radial Distribution Function (RDF) and Coordination Number *vs.* Distance. The first coordination shell is indicated in dashed lines.


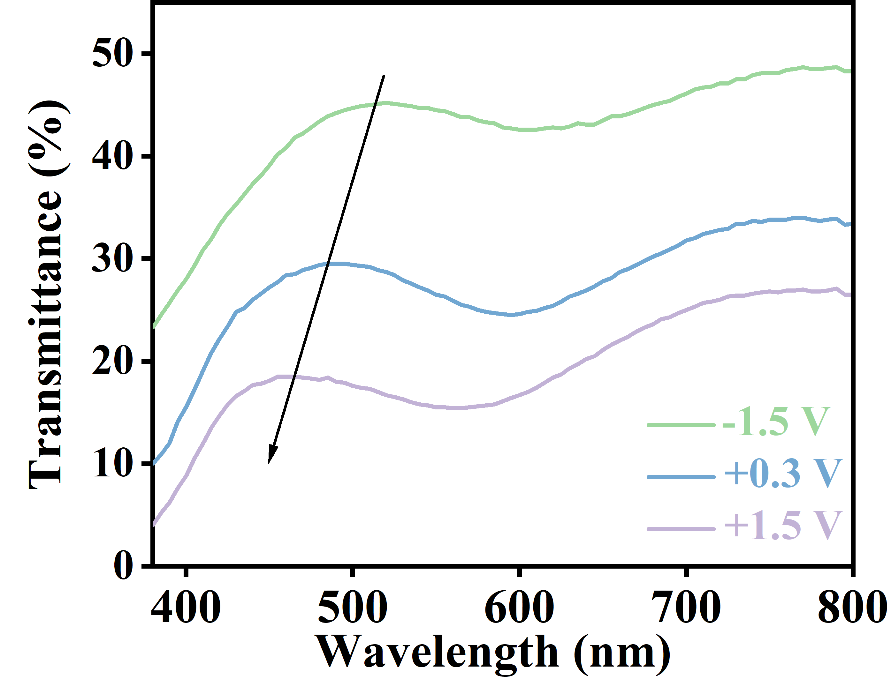


**Figure S25.** *In-situ* transmittance spectra (800-380 nm) of chameleon-FMEDs at -1.5 V, +0.3 V and +1.5 V.


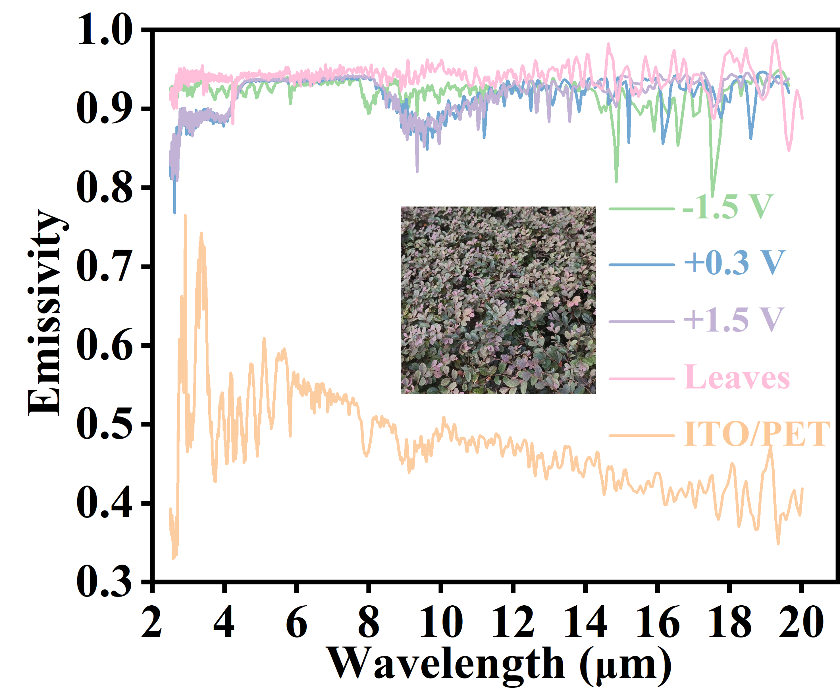


**Figure S26.** Emissivity spectra (2.5-20 μm) of chameleon-FMEDs, ITO/PET and leaves. The insert shows a digital photograph of the leaves.


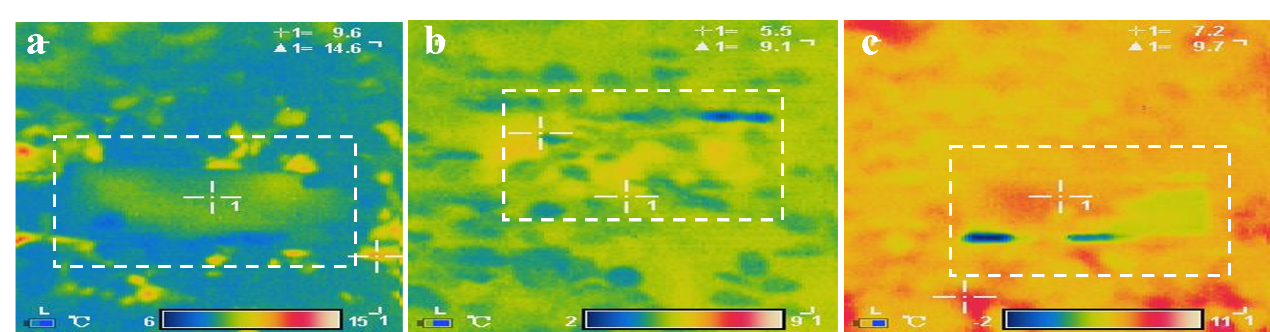


**Figure S27.** Infrared thermal images of Chameleon-like FMED demonstrating infrared camouflage in low temperature (<10 °C).


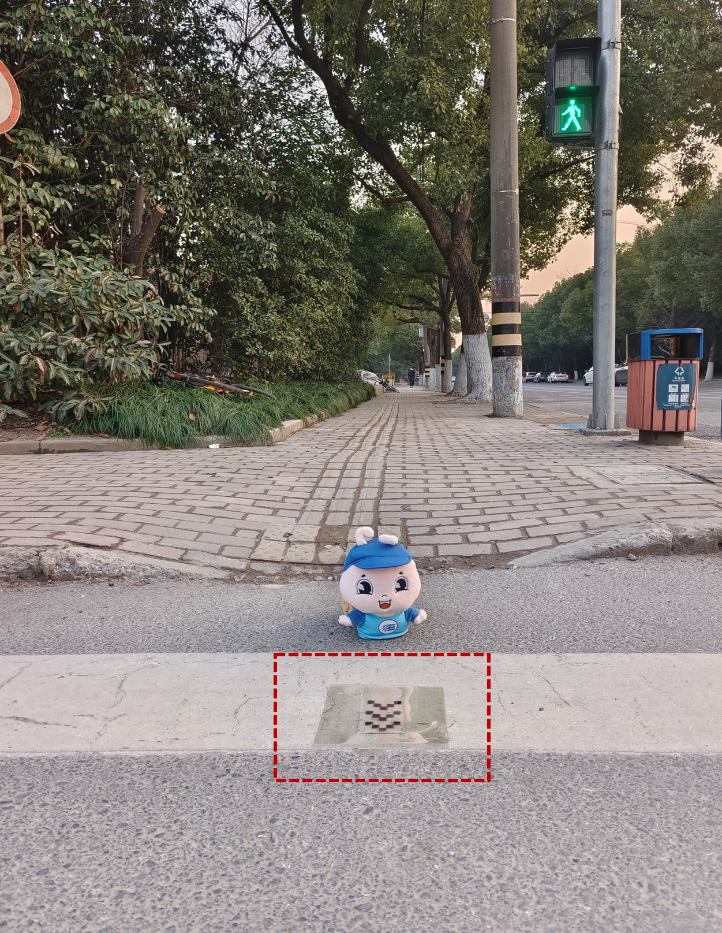


**Figure S28**. Digital photo of our FMED used for road navigation.

**
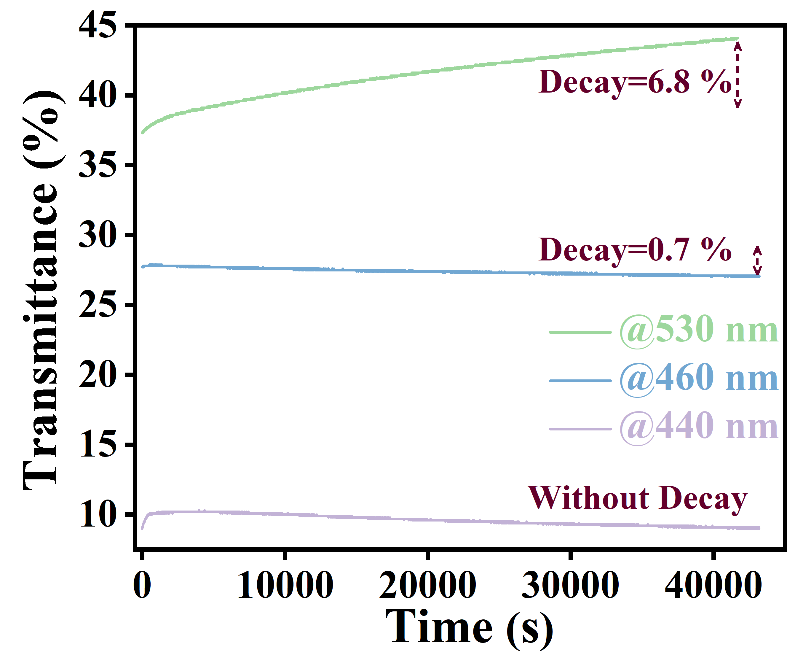
**

**Figure S29.** Memory performance: Variation in transmittance over time at different wavelengths without the external voltages. The displayed patterns in green (530 nm) exhibit only a 6.8% decay after 12 hours, whereas the patterns in bule (460 nm) and purple (440 nm) show a 0.7% decay and no detectable decay, respectively, over the same period.

**4 Supporting Tables**

**Table S1.** The specific bond lengths and dihedral angles of Ni-CAT-1 with and without adsorbed water.

| **Model** | **Ni-O bond length (in pm)** | | | | **α (°)** |
| --- | --- | --- | --- | --- | --- |
| Without adsorbed water | 185 | 188 | 186 | 189 | 2.9 |
| With adsorbed water | 189 | 190 | 191 | 190 | 11.5 |

**Table S2.** The desolvation energy (in eV) of Zn(PC)_4_^2+^ with and without the H_2_O assistance. The detailed reaction mechanism can be found in the Calculation details section.

| **Desolvation step** | **Without adsorbed water** | **With adsorbed water** |
| --- | --- | --- |
| 1^st^ PC | 2.12 eV | -0.88 eV |
| 2^nd^ PC | 2.93 eV | 0.97 eV |
| 3^rd^ PC | 6.22 eV | 1.52 eV |
| 4^th^ PC | 5.04 eV | 2.76 eV |

**References**

1 Kresse, G. & Hafner, J. Ab initio molecular dynamics for liquid metals. *Physical review B* **47**, 558 (1993).

2 Perdew, J. P., Burke, K. & Ernzerhof, M. Generalized gradient approximation made simple. *Physical review letters* **77**, 3865 (1996).

3 Grimme, S., Antony, J., Ehrlich, S. & Krieg, H. A consistent and accurate ab initio parametrization of density functional dispersion correction (DFT-D) for the 94 elements H-Pu. *The Journal of chemical physics* **132** (2010).

4 Nosé, S. A unified formulation of the constant temperature molecular dynamics methods. *The Journal of chemical physics* **81**, 511-519 (1984).

5 Wang, Y. *et al.* Lattice matching growth of conductive hierarchical porous MOF/LDH heteronanotube arrays for highly efficient water oxidation. *Advanced Materials* **33**, 2006351 (2021).

6 Yang, S. *et al.* Unraveling the Electrooxidation Mechanism of 5-(Hydroxymethyl) furfural at a Molecular Level via Nickel-Based Two-Dimensional Metal–Organic Frameworks Catalysts. *ACS Catalysis* **14**, 449-462 (2023).

7 Zhou, W., Lv, S., Liu, X., Li, Y. & Liu, J. A directly grown pristine Cu-CAT metal–organic framework as an anode material for high-energy sodium-ion capacitors. *Chemical communications* **55**, 11207-11210 (2019).

8 Wang, F. *et al.* Turning coordination environment of 2D nickel-based metal-organic frameworks by π-conjugated molecule for enhancing glucose electrochemical sensor performance. *Materials Today Chemistry* **24**, 100885 (2022).

9 Wu, H. *et al.* Conductive metal–organic frameworks selectively grown on laser‐scribed graphene for electrochemical microsupercapacitors. *Advanced Energy Materials* **9**, 1900482 (2019).

10 Dong, J. *et al.* Enhancing the electrocatalytic activity of metal–organic frameworks in the oxygen evolution reaction by introducing high-valent metal centers. *Journal of Materials Chemistry A* **11**, 16683-16694 (2023).

11 Sun, J. *et al.* Conductive Co-based metal–organic framework nanowires: a competitive high-rate anode towards advanced Li-ion capacitors. *Journal of materials chemistry A* **7**, 24788-24791 (2019).

12 Chen, Y. *et al.* Successive storage of cations and anions by ligands of π–d‐conjugated coordination polymers enabling robust sodium‐ion batteries. *Angewandte Chemie* **133**, 18917-18924 (2021).
